# Expression of IL-20 Receptor Subunit β Is Linked to EAE Neuropathology and CNS Neuroinflammation

**DOI:** 10.3389/fncel.2021.683687

**Published:** 2021-09-07

**Authors:** Jacquelyn R. Dayton, Yinyu Yuan, Lisa P. Pacumio, Bryce G. Dorflinger, Samantha C. Yoo, Mariah J. Olson, Sara I. Hernández-Suárez, Moira M. McMahon, Lillian Cruz-Orengo

**Affiliations:** ^1^Department of Anatomy, Physiology and Cell Biology, University of California, Davis, Davis, CA, United States; ^2^Bayer School of Natural and Environmental Sciences, Duquesne University of the Holy Spirit, Pittsburgh, PA, United States; ^3^Department of Molecular and Cell Biology, College of Letters and Science, University of California, Berkeley, Berkeley, CA, United States

**Keywords:** IL-20 subfamily, HCMEC/D3 cells, experimental autoimmune encephalomyelitis, microvessel, MOG-Th1 cells

## Abstract

Considerable clinical evidence supports that increased blood–brain barrier (BBB) permeability is linked to immune extravasation of CNS parenchyma during neuroinflammation. Although BBB permeability and immune extravasation are known to be provoked by vascular endothelial growth factor-A (i.e., VEGF-A) and C-X-C motif chemokine ligand 12 (CXCL12), respectively, the mechanisms that link both processes are still elusive. The interleukin-20 (i.e., IL-20) cytokine signaling pathway was previously implicated in VEGF-mediated angiogenesis and is known to induce cellular response by way of signaling through IL-20 receptor subunit β (i.e., IL-20RB). Dysregulated IL-20 signaling is implicated in many inflammatory pathologies, but it’s contribution to neuroinflammation has yet to be reported. We hypothesize that the IL-20 cytokine, and the IL cytokine subfamily more broadly, play a key role in CNS neuroinflammation by signaling through IL-20RB, induce VEGF activity, and enhance both BBB-permeability and CXCL12-mediated immune extravasation. To address this hypothesis, we actively immunized *IL-20RB^–/–^* mice and wild-type mice to induce experimental autoimmune encephalomyelitis (EAE) and found that *IL-20RB^–/–^* mice showed amelioration of disease progression compared to wild-type mice. Similarly, we passively immunized *IL-20RB^–/–^* mice and wild-type mice with myelin-reactive Th1 cells from either *IL-20RB^–/–^* and wild-type genotype. Host *IL-20RB^–/–^* mice showed lesser disease progression than wild-type mice, regardless of the myelin-reactive Th1 cells genotype. Using multianalyte bead-based immunoassay and ELISA, we found distinctive changes in levels of pro-inflammatory cytokines between *IL-20RB^–/–^* mice and wild-type mice at peak of EAE. We also found detectable levels of all cytokines of the IL-20 subfamily within CNS tissues and specific alteration to IL-20 subfamily cytokines IL-19, IL-20, and IL-24, expression levels. Immunolabeling of CNS region-specific microvessels confirmed IL-20RB protein at the spinal cord microvasculature and upregulation during EAE. Microvessels isolated from macaques CNS tissues also expressed IL-20RB. Moreover, we identified the expression of all IL-20 receptor subunits: IL-22 receptor subunit α-1 (IL-22RA1), IL-20RB, and IL-20 receptor subunit α (IL-20RA) in human CNS microvessels. Notably, human cerebral microvasculature endothelial cells (HCMEC/D3) treated with IL-1β showed augmented expression of the IL-20 receptor. Lastly, IL-20-treated HCMEC/D3 showed alterations on CXCL12 apicobasal polarity consistent with a neuroinflammatory status. This evidence suggests that IL-20 subfamily cytokines may signal at the BBB *via* IL-20RB, triggering neuroinflammation.

## Introduction

Safeguarding CNS homeostasis despite external factors that trigger a deviation from normalcy is paramount for survival. For such reasons, about 500–400 M years ago (Bundgaard and Abbott, 2008) vertebrate animals developed a cellular and molecular “fence,” the blood–brain barrier (BBB), to protect the CNS parenchyma from ions, macromolecules, toxic substances, pathogens, circulating leukocytes, etc. (Daneman and Prat, 2015). Not surprisingly, during BBB disruption the CNS parenchyma becomes susceptible to a myriad of toxic exposures, infection, and inflammation. Thus, dysfunctional BBB is a prominent pathologic feature in many neurologic disorders: Alzheimer’s, Parkinson’s, Huntington’s diseases, multiple sclerosis (MS), autism, epilepsy, stroke, trauma, etc., with significant morbidity and mortality worldwide (Acharjee et al., 2013; Lopes Pinheiro et al., 2016; Sweeney et al., 2018; Yan et al., 2018; Bokobza et al., 2019; Brambilla, 2019; Greene et al., 2019; O’Callaghan and Miller, 2019; Almalki et al., 2021). Currently, its contribution to COVID-19 neurologic involvement is also being studied (Baig et al., 2020; Buzhdygan et al., 2020; Conde Cardona et al., 2020; De Felice et al., 2020; Keller et al., 2020). Brain microvasculature endothelial cells (BMECs) within the neurovascular unit (NVU), conform the BBB (Daneman and Prat, 2015). BMEC are coupled by a peculiar architecture of tight and adherens junctions, adhesion plaques, and “peg-socket junctions,” that are critical for CNS homeostasis (Daneman and Prat, 2015). BMEC properties are induced and maintained by critical interactions with other cells, primarily pericytes, and the surrounding astroglia end-foot processes (Daneman and Prat, 2015). One of the most peculiar features of BBB microvasculature is polarity, i.e., the asymmetrical expression pattern of proteins localized on the luminal or abluminal membrane of BMEC which has been related to both normal function and pathology (Cornford and Hyman, 2005).

The C-X-C motif chemokine ligand 12 (CXCL12) polarity impacts transit to CNS parenchyma of CD4+ lymphocytes co-expressing CXCR4+ (CXCR4+ lymphocytes) (Cruz-Orengo et al., 2011). CXCL12 is normally found along BMEC abluminal surfaces where it regulates lymphocyte access to the CNS parenchyma, acting as a BBB “immune-gate-keeper” (Cruz-Orengo et al., 2011; Williams et al., 2014). During CNS neuroinflammation, CXCL12 loses its polarity, and this distortion highly correlates with sites of active inflammatory loci as observed in neurotropic virus infection, MS, and experimental autoimmune encephalomyelitis (EAE) (Cruz-Orengo et al., 2011; Williams et al., 2014). One mechanism that triggers distortion of CXCL12 polarity is binding to another chemokine receptor, CXCR7. Antagonizing CXCR7-mediated sequestration of CXCL12 prevents its relocation to luminal surfaces, thus halting CXCR4+ lymphocyte extravasation (Cruz-Orengo et al., 2011). Another mechanism is S1PR2-mediated alteration of VE-cadherin, which also impacts CXCL12 apicobasal polarity, with consequent changes in immune extravasation and BBB permeability (Cruz-Orengo et al., 2014; Daniels et al., 2014). Although cytokines can modulate this process, e.g., IL-1β *via* IL-1R, the full contribution of many other cytokines in this process is not well established (McCandless et al., 2009).

A new pleiotropic interleukin-20 (IL-20) subfamily of cytokines, member of the IL-10 super-family, had been characterized as a pro-inflammatory in the context of other immune-mediated diseases (Rutz et al., 2014; Chen et al., 2018). IL-20 was first identified as a keratinocyte autocrine inflammatory mediator (Blumberg et al., 2001). The IL-20 subfamily includes IL-19, IL-20, and IL-24 cytokines and two IL-20 receptors (Dumoutier et al., 2001; Parrish-Novak et al., 2002). IL-20 receptor Type 1, heterodimer of IL-20 receptor subunit α (IL-20RA) and IL-20 receptor subunit β (IL-20RB), and IL-20 receptor Type 2, heterodimer of IL-20RB and IL-22 receptor subunit α-1 (IL-22RA1) (Logsdon et al., 2012; Rutz et al., 2014). The three cytokines can signal *via* IL-20 receptor Type 1 but only IL-20 and IL-24 can signal *via* IL-20 receptor Type 2 (Logsdon et al., 2012; Rutz et al., 2014). Noteworthily, IL-20 had been implicated in numerous immune-mediated pathologies, such as asthma, psoriasis, rheumatoid arthritis (RA), spondyloarthritis, atherosclerosis, diabetes, lupus nephritis, enthesitis, inflammatory bowel disease, and ulcerative colitis (Blumberg et al., 2001; Hsu et al., 2006; Sa et al., 2007; Kragstrup et al., 2008, 2017; Wolk et al., 2008, 2009a,b; Ouyang et al., 2011; Rutz et al., 2014; Hsu and Chang, 2017; Senolt et al., 2017; Chen et al., 2018). Additionally, targeting IL-20 signaling has resulted in a promising therapeutic approach against RA, diabetic nephropathy, osteoarthritis, psoriasis, and cancer (Hsu and Chang, 2010, 2014, 2017; Wang et al., 2012; Chiu et al., 2014; Hsu et al., 2015, 2016, 2017a,b; Zhang et al., 2017).

Remarkably, IL-20 signaling has been in implicated to angiogenesis induced by vascular endothelial growth factor-A (VEGF-A) (Hsieh et al., 2006; Hsu et al., 2006; Wolk et al., 2008, 2009a,b; Hammer et al., 2009; Baird et al., 2011; Ouyang et al., 2011; Han et al., 2016; Kako et al., 2016; Autieri, 2018). More specifically, IL-20 induces phosphorylation of ERK1/2, p38, and JNK and consequently transcription of VEGF in human umbilical vein cells and microvascular endothelial cells (HUVEC, HMEC, respectively) resulting in cell proliferation (Hsieh et al., 2006). It also induces phosphorylation of Erk1/2, PI3K, and mTOR in lymphatic endothelial cells triggering cell migration and tube formation (Hammer et al., 2009). However, dysregulation of IL-20 in non-small cell lung cancer (NSCLC) exerts anti-angiogenic effects by down-regulation of COX-2 (Baird et al., 2011). Also, IL-19 has also been linked to VEGF-mediated angiogenesis by stimulating STAT3 activity in aortic rings (Kako et al., 2016). The fact that IL-20 subfamily cytokines are implicated in VEGF-mediated angiogenesis is relevant because astrocyte-derived VEGF-A enhances BBB permeability during CNS inflammation, *via* VEGF receptor 2 (VEGFR-2) (Argaw et al., 2009, 2012; Davis et al., 2010; Spampinato et al., 2017; Michinaga and Koyama, 2019). Pericytes have been recently characterized as key modulators of VEGF-A autocrine and paracrine actions, especially during neuroinflammatory conditions (Franco et al., 2011; Salmeri et al., 2013; Caporarello et al., 2018; Yang et al., 2018). Additionally, multiple studies shown that (1) microglia and astrocytes are sources and targets of IL-19; (2) mixed glial cells and astrocytes are sources of IL-20 and IL-24, respectively, and (3) microglia and astrocytes are targets to IL-20 and IL-24 (Cooley et al., 2014; Nikfarjam et al., 2014; Burmeister and Marriott, 2018; Burmeister et al., 2019a,b). Furthermore, strong evidence supports the notion that VEGF-mediated enhancement of BBB permeability is linked to immune extravasation (Suidan et al., 2010, 2012; Argaw et al., 2012; Rodrigues and Granger, 2015; Suzuki et al., 2016; Michinaga and Koyama, 2019). Although signaling of subfamily IL-20 is well documented for many inflammatory and autoimmune disorders, its contribution to the pathogenesis of neuroinflammatory disorders has not been reported (Burmeister and Marriott, 2018).

Here, using a canonical neuroinflammatory exemplar, EAE, a murine model for MS, we demonstrate that CNS expression of IL-20 subfamily has a role in neuroinflammation. First, we showed that *IL-20RB^–/–^* mice exhibited ameliorated disease severity compared to wild-type mice and *IL-20^+/–^* mice. Disease amelioration of *IL-20RB^–/–^* mice correlated with a decrease of perivascular infiltrates as assessed by spinal cord histology. This is further validated when *IL-20RB^–/–^* mice submitted to passive immunization with myelin-reactive Th1 cells from either *IL-20RB^–/–^* or wild-type donors displayed the same response. We also showed the presence of IL-20 subfamily cytokines and IL-20RB within the murine CNS by ELISA and microvessels immunolabeling, respectively. Notably we showed expression of IL-20RB in macaques CNS microvessels, plus IL-22RA1, IL-20RA, and IL-20RB in microvessels isolated from human CNS tissues. Moreover, we also shown increase of IL-20 receptor subunits on IL-1β-treated human cerebral microvasculature endothelial cells (HCMEC/D3) and a shift in CXCL12 distribution on with IL-20 stimulation. Together, these data support that IL-20 subfamily plays a key role in neuroinflammation. Further inquiry may confirm this subfamily as putative regulator of VEGF-mediated enhancement of BBB permeability and CXCL12-dependent immune extravasation.

## Materials and Methods

### Animals

*IL-20RB^–/–^* were generated by Lexicon Pharmaceutical as previously described (Zheng et al., 2008; Myles et al., 2013). *IL-20^+/–^* were generated by the UC Davis Mouse Biology Program [C57BL/6N-Il20tm1(KOMP)Vlcg] and C57BL/6 mice were initially purchased from The Jackson Laboratories (Sacramento, CA, United States). All mice were breed in our vivarium and were maintained in pathogen free conditions. The animal studies were reviewed and approved by the University of California, Davis (UC Davis) Institutional Animal Care and Use Committee. Likewise, studies were performed in compliance with such guidelines. Tissues from rhesus macaques were obtained from the California National Primate Research Center Pathology Department (NIH P51OD011107). These macaques were humanely euthanized for the sake of their welfare for non-neurological health issues.

### Human Post-mortem Brain Biopsies

Human brain tissues were provided by the NIH NeuroBioBank (NBB) in compliance with the guidelines of the NBB Brain and Tissue Repositories Material Transfer Agreement (MTA) and UC Davis (IRB) Administration (NBB MTA #1832 and IRB ID #1674251-1, respectively). Post-mortem biopsies were obtained from three healthy females, ages 31 and 32, with non-neurological health issues at the time of death, and certified negative pathology report for neurologic disease.

### Induction of Experimental Autoimmune Encephalomyelitis by Active Immunization

Active immunization was performed on 10 weeks-old male and female mice, *IL-20RB^–/–^*, *IL-20^+/–^*, wild-type, and C57BL/6 with myelin oligodendrocyte glycoprotein (MOG) following standard protocols (Cruz-Orengo et al., 2014). Briefly, naïve C57BL/6 mice were immunized subcutaneously (s.q.) with 50 μg msMOGp35-55 (GenScript, Piscataway, NJ, United States) and 500 μg *Mycobacterium tuberculosis* (Mtb) H37Ra peptide emulsified in Freund’s adjuvant (a.k.a. complete Freund’s adjuvant, CFA, both reagents from Difco Laboratories, Detroit, MI, United States). Mice were injected, intraperitoneally (i.p.), with 300 ng pertussis toxin (List Biological Laboratories, Campbell, CA, United States) at the time of immunization and 2 days after. Sham immunizations for control mice were performed by s.q. injection of equal amounts of Mtb, within the CFA; and i.p. injection of 0.9% injectable saline.

Starting 7 days post-immunization (dpi), immunized mice were monitored for clinical manifestations of EAE by following their body weight and graded for disease progression with the following score system: (1) tail flaccidity; (2) hindlimb paresis; (3) hindlimb paralysis; (4) forelimb paresis; and (5) moribund or dead. Mice were monitored until 28 dpi at which point were humanely euthanized.

### Passive Immunization for EAE Induction

Immunization was performed on 10 weeks-old male and female mice, *IL-20RB^–/–^* mice, and wild-type mice (12 mice per genotype), with MOG emulsion as described for active immunization, except those mice which did not receive pertussis toxin (Cruz-Orengo et al., 2014). Immunized MOG-Th1 “donor” mice were humanely euthanized at 12 dpi for spleen and lymph nodes (inguinal, brachial, axillary, and cervical) harvesting. Briefly, lymphoid organs were washed with RPMI (Gibco, Waltham, MA, United States), supplemented with 5% FBS (Gibco), 1% penicillin/streptomycin (P/S, MilliporeSigma, Burlington, MA, United States), 0.04% β-mercaptoethanol (2βME, Acros Organics, Fair Lawn, NJ, United States), 25 mM HEPES (Gibco), and 2 mM L-glutamine (MilliporeSigma), and scrunched thru a 70 μm cell strainer using a 5 ml syringe plunger. Then, cells were spun at 1100 rpm for 8 min at 4°C. After decanting supernatant, cells were resuspended and incubated in ACK lysing buffer (Gibco) at room temperature for 5 min and spun as before. Cells were resuspended, twice, in RPMI supplemented with 10% FBS, 1% P/S, 2βME, 25 mM HEPES, 2 mM L-glutamine, 1 mM sodium pyruvate (Life Technologies, Waltham, MA, United States), and 0.1 mM NEAA (Life Technologies), and spun as before. After second wash, MOG-Th1 cells were resuspended in same media at 5.0 × 10^6^ MOG-Th1 cells/ml and supplemented with 20 μg/ml msMOGp35-55 (GenScript) and 25 ng/ml IL-12 p70 (Invitrogen, Waltham, MA, United States). MOG-Th1 cells were loaded into T25 flasks and incubated at 37°C, 5–7% CO_2_, for 72 h. After incubation, MOG-Th1 cells were washed twice in RPMI supplemented with 5% FBS, 1% P/S, 0.04% 2βME, 25 mM HEPES, and 2 mM L-glutamine, and spun 1100 rpm for 8 min at 4°C. After, second wash MOG-Th1 cells were resuspended in complete 1× HBSS (Gibco) at a concentration of 10^7^ MOG-Th1 cells/ml. Then, MOG-Th1 were adoptively transferred to naïve recipients, 10 weeks-old male and female, *IL-20RB^–/–^* mice, and wild-type mice, *n* = 8, by injecting i.p. 100 μl of the cell suspension, equivalent to 10^6^ MOG-Th1 cells, following the donor → host scheme: wild-type → wild-type mouse, wild-type → *IL-20RB^–/–^* mouse, *IL-20RB^–/–^* → wild-type mouse, and *IL-20RB^–/–^* → *IL-20RB^–/–^* mouse. Host mice started to be monitored 5 days post-transfer (dpt) for disease progression as described for active immunization, and humanely euthanized at 28 dpt.

### Neutralizing Monoclonal Antibody Treatment *in vivo*

A cohort of actively immunized C57BL/6J, 10 weeks-old male and female mice with either MOG or CFA alone (control) were used as described for active immunization. At 14 dpi, sham-immunized were randomly assigned for either neutralizing rat monoclonal antibody-IL-20 (MAB-IL20) or rat IgG_2B_ isotype (control) treatments, *n* = 4 (MAB12043 and MAB0061, R&D Systems, Minneapolis, MN, United States). MOG-immunized mice were randomly assigned for either treatment, *n* = 8, when they reach a score of 2 (hindlimb paresis). Animals received 10 mg/kg BW of either MAB-IL20 or IgG_2B_ in 200 μl *via* tail vein injection for 10 consecutive days. Sham-immunized mice were euthanized for histological analysis at the completion of treatment. Actively immunized mice were daily monitored for EAE progression as described above and humanely euthanized at 35 dpi.

### Histological Analyses

Detection of spinal cord perivascular infiltrates along white matter was conducted at peak of disease of wild-type mice and *IL-20RB^–/–^* mice with EAE (Cruz-Orengo et al., 2011, 2014). Sham-immunized mice treated with either MAB-IL20 or IgG_2B_ were euthanized to examine for any signs of tissue inflammation or any other abnormality that could be attributed to the use of these biologicals. Deeply anesthetized mice, *n* = 4, were intracardially perfused with 1× PBS followed by fixation with 4% PFA. Then, spinal cords of wild-type mice and *IL-20RB^–/–^* mice with EAE were harvested and post-fixed in 4% PFA in PBS overnight at 4°C. Likewise, lung, skin, knees, gastrointestinal tract (stomach, small/large intestines, and liver) kidney, adrenal gland, spleen, and lymphoid organs (cervical, axillary, and inguinal) were harvested from sham-immunized C57BL/6J treated with either MAB-IL20 or IgG_2B_. Tissues were cryoprotected in 30% sucrose in PBS at 4°C until ready to be embedded for cryosectioning. Embedded tissues were sectioned 8 μm thick over positively charged glass slides. Spinal cord tissue slides were stained with H&E to determine the number of perivascular infiltrates along ventromedial white matter (VMWM), ventral column white matter (VCWM), and ventrolateral white matter (VLWM) white matter (Cruz-Orengo et al., 2011, 2014). All other tissue slides from sham-immunized mice were stained with H&E to assess for any traces of inflammation.

### CNS Detection of Pro-inflammatory and IL-20 Subfamily Cytokines

Expression of IL-19 (88-50320-22, Thermo Fisher Scientific, Waltham, MA, United States), IL-20, IL-24, TNFα, IFNγ, and IL-10 cytokines were determined by ELISA (DY1204, DY2786, DY410, DY485, and DY417, R&D Systems). Pro-inflammatory cytokines known to be relevant during neuroinflammation were detected using a custom multianalyte bead-based immunoassay (LEGENDplex^TM^, BioLegend, San Diego, CA, United States). The analytes included were IL-1β, IL-2, IL-4, IL-6, IL-10, IL-12p70, IL-17A, IL-23, IFN-γ, GM-CSF, TGF-β1, and TNF-α, and trophic factor VEGF. Briefly, deeply anesthetized mice, *n* = 4 per group, were quickly decapitated and CNS tissues were harvested. Tissues were homogenized in 1× PBS supplemented with proteases and phosphatases inhibitors (A32955, Pierce-Thermo Fisher Scientific). After homogenization, tissue lysates were tested following manufacturer instructions. ELISA samples were analyzed using the Synergy H1-plate reader and Gen5 Data Analysis Software (BioTek Instruments, Winooski, VT, United States). LEGENDplex samples were analyzed using the BD LSRFortessa^TM^ (BD Biosciences, Franklin Lakes, NJ, United States) and LEGENDplex software (Lehmann et al., 2017).

### IL-20 Receptor Subunits Protein Expression in CNS Microvasculature

Four per group were euthanized for CNS region-specific microvessels isolation (Dayton et al., 2019; Yuan et al., 2020). Deeply anesthetized mice were decapitated, and CNS tissues quickly dissected and kept in ice-cold 1% 1 M HEPES in HBSS (HEPES/HBSS, Gibco). Then the meninges, thalamus, choroid plexus, and major caliber blood vessels were excised under a stereoscope. Brain cortex, cerebellum, brainstem, and spinal cord were individually minced with a single-edge blade and homogenized in 5 ml of HEPES/HBSS in a Potter-Elvehjem glass tissue grinder. Resulting homogenates were spun at 2000 × *g* for 10 min at 4°C. Then, pellets were resuspended in 10 ml 18% dextran (MilliporeSigma) in HEPES/HBSS and spun at 4400 × *g* for 15 min at 4°C. Myelin interphase were carefully decanted and microvessels pellets were resuspended in 1 ml 1% bovine serum albumin (BSA, MilliporeSigma) in HEPES/HBSS. Myelin debris were further removed by loading the microvessels lysates over 100 μm cell strainers and rinsed with 10 ml 1% BSA/HEPES/HBSS. Eluted microvessels were then collected by pouring over a 20 μm nylon net filter (MilliporeSigma) previously equilibrated with 5 ml 1% BSA/HEPES/HBSS. After rinsing with 10 ml 1% BSA/HEPES/HBSS the nylon net filters were transferred into beakers with ice-cold 10 ml 1% BSA/HEPES/HBSS and gently shaken to release the microvessels. After centrifuging at 2000 × *g* for 5 min at 4°C, pellets were resuspended in 1 ml of ice-cold 1% BSA/HEPES/HBSS and spun again. Then, pellets were resuspended in 200–1000 μl 1× PBS, depending on pellet size, prior to loading on chamber slides coated with Cultrex^®^ Poly-D-Lysine (R&D Systems).

Immunodetection of IL-20RB within CNS parenchyma microvessels was performed with IL-20RB (BAF1788, R&D Systems) and co-labeling with platelet endothelial cell adhesion molecule 1 (PECAM1, CD31, 550274, BD, Franklin Lakes, NJ, United States), neural/glial antigen 2 (AB5320, MilliporeSigma), and aquaporin-4 (AQP4, MA5-24587, Thermo Fisher Scientific). Chambers slides with freshly isolated microvessels were fixed in 4% PFA for 30 min at room temperature. After washing fixative with PBS, microvessels were permeabilized and blocked in 0.1% Triton X-100/10% BSA in PBS for 60 min at 37°C. Slides were incubated with primary antibody in 0.1% Triton X-100/5% BSA in PBS overnight at 4°C. After washing three times for 5 min in PBS, microvessels were incubated with secondary antibodies diluted in PBS for 2 h at room temperature (Molecular Probes-Thermo Fisher Scientific). After washing secondary antibodies, slides were cover slipped with ProLong^®^ Diamond Antifade Mountant with DAPI (Thermo Fisher Scientific) to detect nuclei. Immunostained sections were visualized on the Leica TCS SP8 STED 3× confocal microscope and LAS X software (Leica Biosystems, Nussloch, Germany).

We isolated CNS region-specific microvessels from rhesus macaque CNS tissues after the modifications previously published (Dayton et al., 2019; Yuan et al., 2020). The most notable differences to the aforementioned protocol were the use of 55 ml Potter-Elvehjem glass tissue grinder aimed by a Wheaton overhead stirrer, the use of 20% dextran/HEPES/HBSS for myelin separation and mouse anti-human CD31 and VEGFR-2 antibodies (BBA7 and MAB3571, R&D Systems). Additionally, we isolated microvessels from human post-mortem brain biopsies. Tissues were obtained from the periventricular white matter (PVWM) region of healthy individuals, *n* = 3, female ages 30–32 years old. Immunolabeling was performed using antibodies against CD31, platelet-derived growth factor receptor β (PDGFRB), IL-20RB, and IL-22RA1 (BBA7, AF1042, BAF1788, and MAB2770, R&D Systems), IL-20RA (ab203196, Abcam, Cambridge, MA, United States), and AQP4 (MA5-24587, Thermo Fisher Scientific).

### IL-1β and IL-20 Cytokines Treatment of Human Cerebral Microvasculature Endothelial Cells *in vitro*

Human cerebral microvasculature endothelial HCMEC/D3 cells were expanded in EBM-2 Basal Medium (EGM-2^TM^ MV Bullet Kit^TM^, Lonza, Walkersville, MD, United States) following manufacturer instructions and previously described (Weksler et al., 2005, 2013; Aaron et al., 2018; Aaron and Gelli, 2020). Then, cells were transferred from T-25 cm^2^ flask to collagen-coated chamber slides at a 0.02 × 10^6^ concentration in a total volume of 500 μl of 1× media (1× media = 500 ml EBM^TM^-2 Basal Medium plus CC-4147 SingleQuots^TM^ Kit to formulate EGM-2MV. SingleQuots has a proprietary formulation containing FBS, hydrocortisone, hFGF-B, VEGF, hR^3^IGF-1, ascorbic acid, hEGF, and gentamicin sulfate/amphotericin). Cells were maintained at 37°C, 5% CO_2_. After 4 days, when cells reached confluency (∼0.8 to 1.0 × 10^6^ cells) media was change to 1× media. At day 7, media was changed to 1/2× media and at day 11 to 1/4× media (dilutions alter FBS, hydrocortisone, hFGF-B, VEGF, hR^3^IGF-1, ascorbic acid, and hEGF concentrations but, not gentamicin sulfate/amphotericin). At day 12, cells were either treated for 24 h with either 1 ng/ml of recombinant human-IL-1β (rhIL-1β) or 10 ng/ml of recombinant human-IL-20 (rhIL-20) (201-LB-005 and 1102-IL-025, R&D Systems) or remained in media absent of cytokine (control).

After cytokine treatment, HCMEC/D3 cells were fixed in ice cold 4% PFA for 10 min. Then, washed for 5 min in PBS and blocked for 15 min in 10% donkey serum/0.1% Triton X-100 in PBS at room temperature. After a 5 min wash in PBS, cells were incubated in primary antibody diluted in blocking solution for 1 h at room temperature. Primary antibodies used: IL-20RB and IL-22RA1 (BAF1788 and MAB2770, R&D Systems), IL-20RA (ab203196, Abcam, Cambridge, MA, United States), type 1 gamma-glutamyltransferase (GGT1, ab55138, Abcam), and CXCL12 (500-P87BGBt, PeproTech, Cranbury, NJ, United States). Then, washed three times for 5 min in 1× PBS and incubated in secondary antibodies, diluted in PBS, for 15 min at room temperature (Molecular Probes-Thermo Fisher Scientific). After washing secondary antibodies, slides were cover slipped with ProLong Diamond Antifade Mountant with DAPI (Thermo Fisher Scientific) to detect nuclei. Immunostained sections were visualized on the Leica TCS SP8 STED 3× confocal microscope and LAS X software (Leica Biosystems, Nussloch, Germany).

### Statistical Analysis

All statistical analysis was done using Prism 9.1.2 (GraphPad Software, La Jolla, CA, United States). *N* value for *in vivo* experiments for clinical scoring were determined in order to reach a power value ∼0.8 (lowering risk of Type 2 error): *n* = 10 for *IL-20RB^–/–^*, *IL-20^+/–^*, and wild-type mice experiments, *n* = 8 for wild-type → wild-type, wild-type → *IL-20RB^–/–^*, *IL-20RB^–/–^* → wild-type, and *IL-20RB^–/–^* → *IL-20RB^–/–^* adoptive transfer experiments, and *n* = 12 for MABIL-20 and IgG_2B_ experiments. Additionally, *n* value has equal number of male and female mice per group. No sex differences were observed in any of the clinical experiments thus, data was compiled for further analysis. Clinical scores and body weight, highest and cumulative scores, and disease onset for *IL-20RB^–/–^*, wild-type mice comparison and adoptive transfer were analyzed *via* non-parametric Kruskal–Wallis followed by Dunn’s *post hoc* test. Clinical scores and body weight, highest and cumulative scores, and disease onset for *IL-20^+/–^* and MABIL-20 vs. IgG_2B_ were done by unpaired *t*-test. A *p* < 0.05 was deemed significant for all statistical analysis.

For histology (H&E), ELISA, multianalyte bead-based immunoassay, murine CNS microvessels isolation, and immunolabeling analyses, *n* = 4 (equal number of male and female mice per group). For microvessels isolation and immunolabeling of murine, *n* = 4, 25 microvessels per sample, and rhesus macaques and human post-mortem biopsies, *n* = 3, 50 microvessels per sample. *In vitro* HCMEC/D3 cells histogram analysis of 25 cells per group, *n* = 3, were done in triplicates. Data obtained from ELISA, multianalyte bead-based immunoassay, quantitation of macaque and human microvessels immunolabeling and *in vitro* HCMEC/D3 experiments were analyzed *via* one-way ANOVA followed by Sidak’s *post hoc* test. Data from histology and murine microvessels immunolabeling was analyzed by unpaired *t*-test. A *p* < 0.05 was deemed significant for all statistical analysis.

## Results

### Absence of IL-20RB Gene Expression Ameliorates EAE Progression

**FIGURE 1:**
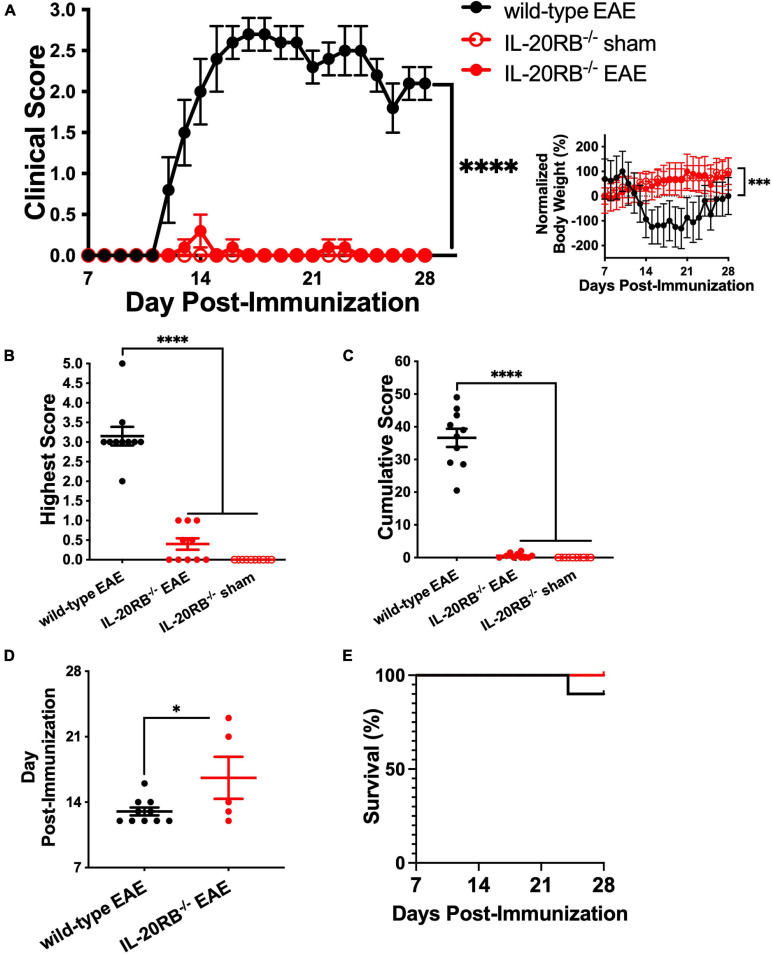
Genetic ablation of IL-20RB ameliorates EAE. Ten week-old *IL-20RB^–/–^* mice and wild-type mice, male and female, were actively immunized for EAE (red circle and black circle, respectively), using *IL-20RB^–/–^* sham-immunized mice (red outline) as control (*n* = 10). Clinical score, normalized body weight, highest and cumulative scores from 7 to 28 dpi were analyzed by non-parametric Kruskal–Wallis followed by Dunn’s *post hoc* test comparing actively immunized *IL-20RB^–/–^* mice with wild-type mice, and actively immunized *IL-20RB^–/–^* mice with sham-immunized *IL-20RB^–/–^* mice **(A–C)**. Difference in clinical score between *IL-20RB^–/–^* mice with EAE (mean ± SEM: 0.03 ± 0.02) and wild-type mice (mean ± SEM: 1.72 ± 0.22) with EAE showed extreme statistical significance, *p* < 0.0001, while no difference was observed when compared with sham-immunized *IL-20RB^–/–^* mice (mean ± SEM: 0.00 ± 0.00) **(A)**. Likewise, difference in normalized body weight was highly significant between *IL-20RB^–/–^* mice with EAE (mean ± SEM: 51.82 ± 6.81) and wild-type mice (mean ± SEM: –39.77 ± 16.21) with EAE *p* < 0.001 but not when compared to sham-immunized *IL-20RB^–/–^* mice (mean ± SEM: 59.24 ± 5.40) (**A**, insert). Highest **(B)** and cumulative scores **(C)** analysis showed extreme statistical significance for *IL-20RB^–/–^* mice with EAE (mean ± SEM highest score: 0.40 ± 0.15, cumulative score: 0.55 ± 0.23) and wild-type mice with EAE (mean ± SEM highest score: 3.15 ± 0.24, cumulative score: 36.60 ± 2.78), *p* < 0.0001, but not with sham-immunized *IL-20RB^–/–^* mice (mean ± SEM highest score: 0.00 ± 0.00, cumulative score: 0.00 ± 0.00). Analysis of EAE disease onset by unpaired *t*-test for actively immunized *IL-20RB^–/–^* mice with neurologic symptoms (5/10, mean ± SEM: 16.60 ± 2.25) and wild-type mice (mean ± SEM: 13.00 ± 0.42) showed statistical significance, *p* < 0.05 **(D)**. Analysis of percentage of survival was not deemed significant **(E)**. Results are shown as mean ± SEM, ^∗^*p* < 0.05, ^∗∗∗^*p* < 0.001, and ^****^p < 0.0001.

To assess if IL-20 subfamily cytokines signaling through IL-20RB plays a role on EAE neuroinflammation and pathology, active immunization of IL-20RB knock-out mice and wild-type mice was conducted. We followed all groups for EAE disease progression and compared with sham-immunized *IL-20RB^–/–^* mice. When compared to wild-type mice, *IL-20RB^–/–^* mice exhibited a strong suppression of EAE disease progression (Figure 1A). When we analyzed the clinical score and normalized body weight of *IL-20RB^–/–^* mice and wild-type mice with EAE by non-parametric Kruskal–Wallis followed by Dunn’s *post hoc* test, it showed a significant statistical difference between the mean of both groups, *p* < 0.0001 and *p* < 0.001, respectively (Figure 1A). However, no statistically significant difference was achieved when comparing *IL-20RB^–/–^* mice sham-immunized with the actively immunized mice (Figure 1A). When we analyzed the highest score achieved as an indicator of disease severity, we found that there was an extreme degree of statistical significance between *IL-20RB^–/–^* mice and wild-type mice with EAE, *p* < 0.0001, but not with *IL-20RB^–/–^* mice that were sham-immunized (highest score mean ± SEM for wild-type mice and *IL-20RB^–/–^* mice with EAE and *IL-20RB^–/–^* sham-immunized: 3.15 ± 0.24, 0.40 ± 0.15, and 0.0 ± 0.0, Figure 1B). Likewise, the comparison of cumulative clinical scores was extremely significant for *IL-20RB^–/–^* mice compared to wild-type mice with EAE, *p* < 0.0001, but no difference with sham-immunized mice (cumulative score mean ± SEM for wild-type mice with EAE = 36.60 ± 2.78, *IL-20RB^–/–^* mice with EAE = 0.55 ± 0.23, and *IL-20RB^–/–^* mice sham-immunized = 0.0 ± 0.0, Figure 1C). *IL-20RB^–/–^* mice actively immunized showed delay of disease onset compared to wild-type mice with EAE that is statistically significant, *p* < 0.05 (dpi of EAE onset mean ± SEM for wild-type with EAE = 13.00 ± 0.42, and *IL-20RB^–/–^* mice with EAE = 16.60 ± 2.25, Figure 1D). Notably none of the actively immunized *IL-20RB^–/–^* mice that developed neurologic symptoms, 5 out of 10, succumbed to the disease unlike wild-type mice with EAE (Figure 1E). Quantification of perivascular infiltrates in the spinal cord of wild-type mice and *IL-20RB^–/–^* mice showed a statistically significant lower number for *IL-20RB^–/–^* mice (Supplementary Figure 1). These data strongly demonstrate that IL-20RB does play a role in EAE neuropathology.

We also wanted to test our initial hypothesis by active immunization of IL-20 knock-out mice. Homozygosity of *IL-20^–/–^* resulted to be embryonic lethal, therefore we actively immunized *IL-20^+/–^* mice instead. Unfortunately, heterozygosity of *IL-20^+/–^* was not sufficient to ameliorate EAE disease progression. Actively immunized *IL-20^+/–^* mice showed normal EAE disease progression (Supplementary Figure 2). It also suggests that IL-20 may not be the only cytokine of this subfamily involved in this process, and that IL-19 and IL-24 might also contribute to IL-20 receptor role in CNS neuroinflammation.

### IL-20RB Expression at the CNS, Rather Than MOG-Th1 Cells, Plays a Relevant Role in EAE Development

**FIGURE 2:**
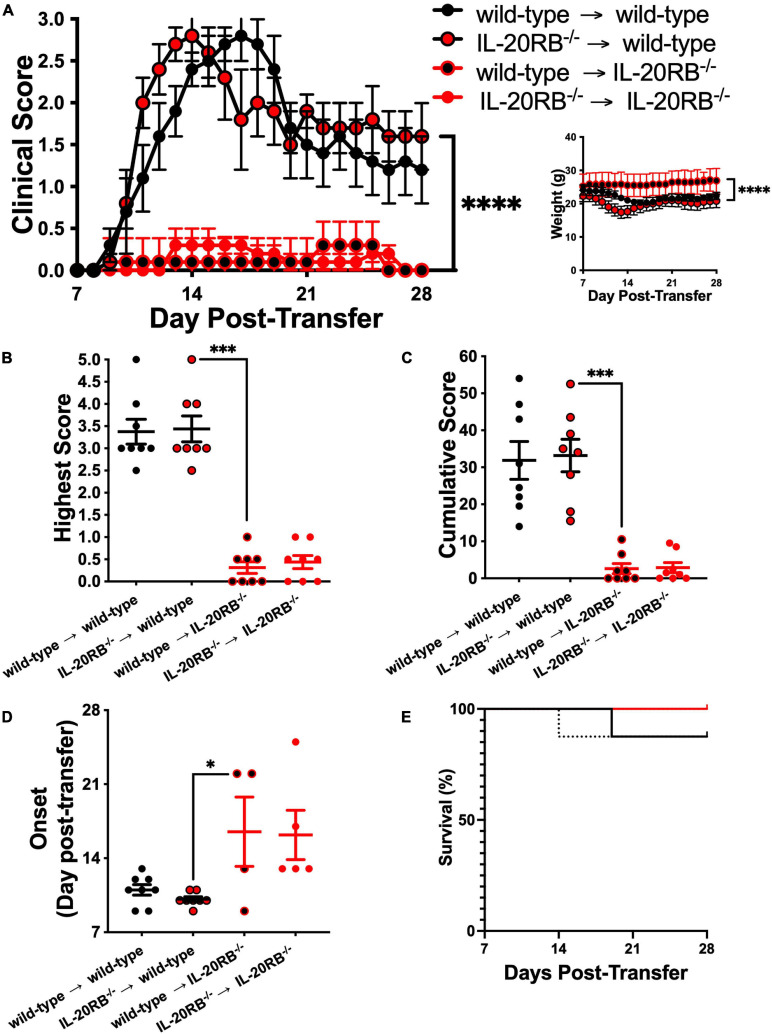
Genotype of MOG-reactive Th1 cells does not contribute significantly to EAE while host genotype determines disease outcome. Ten week-old *IL-20RB^–/–^* mice and wild-type mice, male and female, were passively immunized for EAE with 10^6^ cells of either *IL-20RB^–/–^* MOG-Th1 or wild-type MOG-Th1 cells (*n* = 8). Donor to host mouse groups were the following: wild-type → wild-type mice (black circle), *IL-20RB^–/–^* → wild-type mice (red circle/black outline), wild-type → *IL-20RB^–/–^* mice (black circle/red outline), and *IL-20RB^–/–^* → *IL-20RB^–/–^* mice (red circle). Clinical score, body weight, highest and cumulative scores, and disease onset from 7 to 28 dpi were analyzed by non-parametric Kruskal–Wallis followed by Dunn’s *post hoc* test comparing primarily *IL-20RB^–/–^* → wild-type mice with wild-type → *IL-20RB^–/–^* mice **(A–D)**. Wild-type → wild-type mice (mean ± SEM clinical score: 1.53 ± 0.18, body weight: 21.93 ± 0.25), and *IL-20RB^–/–^* → *IL-20RB^–/–^* mice (mean ± SEM clinical score: 0.13 ± 0.03, body weight: 26.10 ± 0.28) showed outcomes comparable to wild-type mice and *IL-20RB^–/–^* mice with EAE (Figure 1A); but differences in clinical score and body weight between *IL-20RB^–/–^* → wild-type mice (mean ± SEM clinical score: 1.66 ± 0.17, body weight: 20.09 ± 0.28), and wild-type → *IL-20RB^–/–^* mice (mean ± SEM clinical score: 0.11 ± 0.02, body weight: 25.99 ± 0.11) showed extreme statistical significance, *p* < 0.0001 **(A)**. Highest **(B)** and cumulative scores **(C)** analysis showed high statistical significance between *IL-20RB^–/–^* → wild-type mice (mean ± SEM highest score: 3.44 ± 0.29, cumulative score: 33.19 ± 4.41), and wild-type → *IL-20RB^–/–^* mice (mean ± SEM highest score: 0.31 ± 0.13, cumulative score: 2.62 ± 1.37), *p* < 0.001. Analysis of EAE disease onset for mice with neurologic symptoms (wild-type → wild-type mice and *IL-20RB^–/–^* → wild-type mice, 8/8; wild-type → *IL-20RB^–/–^* mice, 4/8, and *IL-20RB^–/–^* → *IL-20RB^–/–^* mice, 5/8) showed statistical significance between *IL-20RB^–/–^* → wild-type mice (mean ± SEM: 10.13 ± 0.23) and wild-type → *IL-20RB^–/–^* mice (mean ± SEM: 16.50 ± 6.07), *p* < 0.05 **(D)**. Again, highest and cumulative scores, and disease onset **(B–D)** of wild-type → wild-type mice (mean ± SEM highest score: 3.38 ± 0.28, cumulative score: 31.88 ± 5.12, disease onset: 11.00 ± 0.50) and *IL-20RB^–/–^* → *IL-20RB^–/–^* mice (mean ± SEM highest score: 0.44 ± 0.15, cumulative score: 2.88 ± 1.38, disease onset: 16.2 ± 2.33) were similar as the ones observed for wild-type mice and *IL-20RB^–/–^* mice with EAE (Figures 1B–D). Analysis of percentage of survival was not deemed significant **(E)**. Results are shown as mean ± SEM, ^∗^*p* < 0.05, ^∗∗∗^*p* < 0.001, and ^****^p < 0.0001.

Once we determined that IL-20RB contributes to EAE neuropathology, we wanted to elucidate if such expression is required for CNS susceptibility or the peripheral immune system production of MOG-reactive Th1 cells. We answered this question by performing adoptive transfer of MOG-reactive Th1 cells obtained from MOG-immunized wild-type mice into naïve *IL-20RB^–/–^* mice, to assess if IL-20RB impacts CNS susceptibility. We also performed adoptive transfer of MOG-Th1 cells from *IL-20RB^–/–^* mice into naïve wild-type mice to identify if IL-20RB contribution is required at the peripheral immune system. The other two adoptive transfer groups, wild-type → wild-type and *IL-20RB^–/–^* → *IL-20RB^–/–^*, were performed as positive control. Notably, wild-type → *IL-20RB^–/–^* mice presented a robust amelioration of EAE progression, like *IL-20RB^–/–^* → *IL-20RB^–/–^* control group (Figure 2A). The opposite was the case for *IL-20RB^–/–^* → wild-type showing EAE disease severity as pronounced as wild-type → wild-type (Figure 2A). Further analysis of the clinical scores by non-parametric Kruskal–Wallis followed by Dunn’s *post hoc* test, showed a significant statistical difference between the mean of, *IL-20RB^–/–^* → wild-type mice with wild-type → *IL-20RB^–/–^* mice, *p* < 0.0001. We also observed that disease severity and cumulative score were lesser for wild-type → *IL-20RB^–/–^* mice than *IL-20RB^–/–^* → wild-type mice and this difference was extremely statistically significant (highest score mean ± SEM for *IL-20RB^–/–^* → wild-type mice = 3.44 ± 0.29, wild-type → *IL-20RB^–/–^* mice = 0.31 ± 0.13, *p* < 0.0001, Figure 2B; cumulative score mean ± SEM for *IL-20RB^–/–^* → wild-type mice = 33.19 ± 4.41, wild-type → *IL-20RB^–/–^* mice = 2.63 ± 1.37, *p* < 0.0001, Figure 2C). Wild-type → *IL-20RB^–/–^* mice also showed a statistically significant delay in disease onset (dpt of EAE onset mean ± SEM for *IL-20RB^–/–^* → wild-type mice = 10.13 ± 0.23, wild-type → *IL-20RB^–/–^* mice = 16.50 ± 3.28, Figure 2D). Although 4 out of 8 wild-type → *IL-20RB^–/–^* mice developed neurologic symptoms, none of them succumbed to EAE unlike *IL-20RB^–/–^* → wild-type mice (Figure 2E). The aforementioned is consistent with our central hypothesis that IL-20 receptors expression at the CNS confers susceptibility to EAE neuropathology, presumably by triggering immune extravasation of MOG-reactive Th1 through the NVU that conforms the BBB.

Another approach we interrogated was neutralization *via* monoclonal antibodies. Of the cytokines within this subfamily, IL-19, IL-20, and IL-24, only IL-20 has a commercially available neutralizing monoclonal antibody (Hsu et al., 2011, 2017a; Chiu et al., 2014; Mayer et al., 2015; Senolt et al., 2015; Kragstrup et al., 2018). Additionally, this antibody had been used *in vivo* in other murine models of inflammatory disorders and RA clinical trials but, not for neuroinflammatory conditions (Hsu et al., 2015; Senolt et al., 2015). We initially sham-immunized, *n* = 4, 10-weeks old C57BL/6J mice, male and female, and treated them with either MABIL-20 or IgG_2B_, 10 mg/kg BW, for 10 days. We perform this assessment as a safety assay, to determine if MABIL-20 could trigger any unwanted inflammatory response. Fortunately, that was not the case and we safely continued with MAB-IL20 treatment during EAE (Supplementary Figure 3). Treatment with either MAB-IL20 or IgG of actively immunized mice, *n* = 8, proceeded as described when they reach a score of 2. However, we did not obtain the anticipated result (Supplementary Figure 4). Since, MABIL-20-treated mice showed a rebound in EAE progression when MABIL-20 treatment ended (Supplementary Figure 4A) we speculated that a higher dose, or earlier therapy initiation, or a prolonged period of treatment might exert therapeutic benefit.

### IL-20 Subfamily Cytokines Are Present at Murine CNS Tissues and IL-20RB Protein Is Expressed in CNS Microvasculature

**FIGURE 3:**
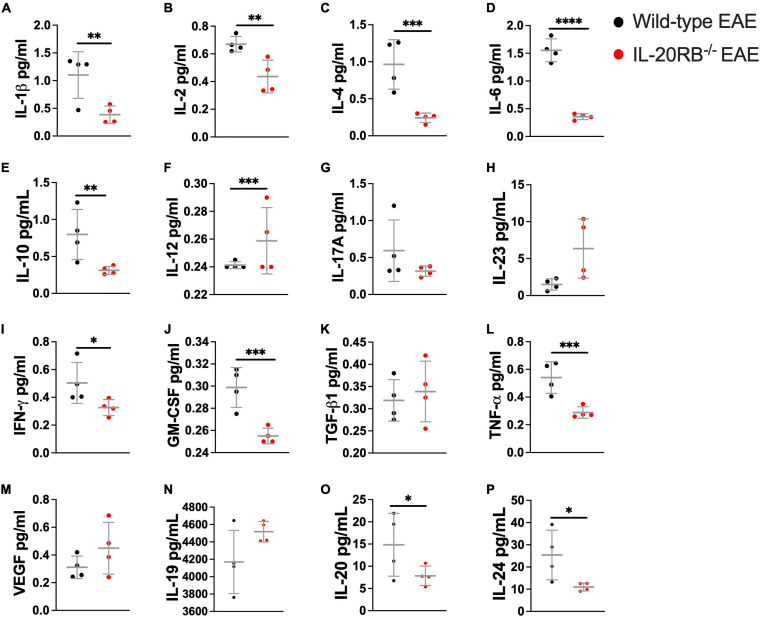
Cytokine expression in the spinal cord of wild-type mice and *IL-20RB^–/–^* mice at peak of EAE. Cytokines and VEGF expression within spinal cord tissues was detected by multianalyte bead-based immunoassay **(A–M)** and ELISA **(N–P)**, *n* = 4, for wild-type mice at peak of EAE (peak, ∼14 dpi) (black circle) and sham-immunized, *IL-20RB^–/–^* mice (red circle), and sham-immunized (sham-immunized not shown). One-way ANOVA analysis followed by Sidak’s *post hoc* test was perform comparing primarily wild-type mice and *IL-20RB^–/–^* mice at peak of EAE and showed that mean differences to be significant for many cytokines: IL-1β (mean ± SEM: 1.10 ± 0.21 vs. 0.39 ± 0.08, *p* < 0.01, **A**), IL-2 (mean ± SEM: 0.67 ± 0.05 vs. 0.44 ± 0.06, *p* < 0.01, **B**), IL-4 (mean ± SEM: 0.96 ± 0.17 vs. 0.24 ± 0.03, *p* < 0.001, **C**), IL-6 (mean ± SEM: 1.36 ± 0.10 vs. 0.36 ± 0.03, *p* < 0.0001, **D**), IL-10 (mean ± SEM: 0.80 ± 0.17 vs. 0.31 ± 0.03, *p* < 0.01, **E**), IL-12 (mean ± SEM: 0.24 ± 0.001 vs. 0.26 ± 0.01, *p* < 0.001, **F**), IFN-γ (mean ± SEM: 0.50 ± 0.07 vs. 0.33 ± 0.03, *p* < 0.05, **I**), GM-CSF (mean ± SEM: 0.30 ± 0.01 vs. 0.26 ± 0.003, *p* < 0.001, **J**), TNF-α (mean ± SEM: 0.54 ± 0.06 vs. 0.29 ± 0.02, *p* < 0.001, **L**), IL-20 (mean ± SEM: 14.83 ± 3.54 vs. 7.85 ± 1.09, *p* < 0.05, **O**), and IL-24 (mean ± SEM: 25.43 ± 5.59 vs. 11.04 ± 0.91, *p* < 0.05, **P**). Results are shown as mean ± SEM, ^∗^*p* < 0.05, ^∗∗^*p* < 0.01, ^∗∗∗^*p* < 0.001, and ^****^p < 0.0001.

To identify the expression of IL-20 subfamily cytokines murine CNS we measured cytokine expression by ELISA, *n* = 4, in wild-type mice and *IL-20RB^–/–^* mice, sham-immunized and actively immunized (Figure 3 and Supplementary Figures 5–7). We also examined the CNS and serum expression of cytokines IL-1β, IL-2, IL-4, IL-6, IL-10, IL-12p70, IL-17A, IL-23, IFN-γ, GM-CSF, TGF-β1, and TNF-α, and trophic factor VEGF by multianalyte bead-based immunoassay (Figure 3 and Supplementary Figures 5–8). Analyses were performed at peak of neurologic disability for EAE, ∼14 dpi. We obtained detectable levels of IL-19, IL-20, and IL-24 cytokines for all mice groups and CNS tissues under investigation (Figure 3 and Supplementary Figures 5–7). We observed differences in expression levels at the spinal cord between wild-type mice and *IL-20RB^–/–^* mice with EAE for most of these cytokines (Figure 3). One-way ANOVA analysis followed by Sidak’s *post hoc* test showed that these differences were statistically significant for IL-1β (mean ± SEM wild-type mice vs. *IL-20RB^–/–^* mice: 1.10 ± 0.21 vs. 0.38 ± 0.08, *p* < 0.01), IL-2 (mean ± SEM wild-type mice vs. *IL-20RB^–/–^* mice: 0.67 ± 0.03 vs. 0.44 ± 0.06, *p* < 0.01), IL-4 (mean ± SEM wild-type mice vs. *IL-20RB^–/–^* mice: 0.96 ± 0.17 vs. 0.24 ± 0.03, *p* < 0.001), IL-6 (mean ± SEM wild-type mice vs. *IL-20RB^–/–^* mice: 1.55 ± 0.10 vs. 0.36 ± 0.03, *p* < 0.0001), IL-10 (mean ± SEM wild-type vs. *IL-20RB^–/–^* mice: 0.80 ± 0.17 vs. 0.31 ± 0.03, *p* < 0.01), IL-12p70 (mean ± SEM wild-type mice vs. *IL-20RB^–/–^* mice: 0.24 ± 0.001 vs. 0.26 ± 0.001, *p* < 0.001), IFN-γ (mean ± SEM wild-type mice vs. *IL-20RB^–/–^* mice: 0.50 ± 0.07 vs. 0.33 ± 0.03, *p* < 0.05), GM-CSF (mean ± SEM wild-type mice vs. *IL-20RB^–/–^* mice: 0.30 ± 0.01 vs. 0.26 ± 0.003, *p* < 0.001), TNF-α (mean ± SEM wild-type mice vs. *IL-20RB^–/–^* mice: 0.54 ± 0.06 vs. 0.29 ± 0.03, *p* < 0.001), IL-20 (mean ± SEM wild-type mice vs. *IL-20RB^–/–^* mice: 14.83 ± 3.53 vs. 7.85 ± 1.09, *p* < 0.05), and IL-24 (mean ± SEM wild-type mice vs. *IL-20RB^–/–^* mice: 25.43 ± 5.59 vs. 11.04 ± 0.91, *p* < 0.05). Other CNS regions showed changes in cytokine expression between wild-type mice and *IL-20RB^–/–^* mice but not as pronounced as the spinal cord, where EAE neuropathology and neuroinflammation is preponderant (mean ± SEM wild-type mice vs. *IL-20RB^–/–^* mice: Supplementary Figure 5, cortex: IL-23 5.90 ± 1.08 vs. 2.32 ± 0.51, *p* < 0.05, GM-CSF 0.27 ± 0.01 vs. 0.24 ± 0.002, *p* < 0.01, and TNF-α 0.30 ± 0.01 vs. 0.26 ± 0.01, *p* < 0.05, and IL-24 45.71 ± 2.83 vs. 8.40 ± 0.79, *p* < 0.0001. Supplementary Figure 6, cerebellum: GM-CSF 0.26 ± 0.007 vs. 0.24 ± 0.00, *p* < 0.05. Supplementary Figure 7, brainstem: GM-CSF 0.28 ± 0.004 vs. 0.25 ± 0.004, *p* < 0.01, and TNF-α 0.33 ± 0.02 vs. 0.27 ± 0.004, *p* < 0.01). No differences were observed in cytokines serum expression levels (Supplementary Figure 8).

**FIGURE 4:**
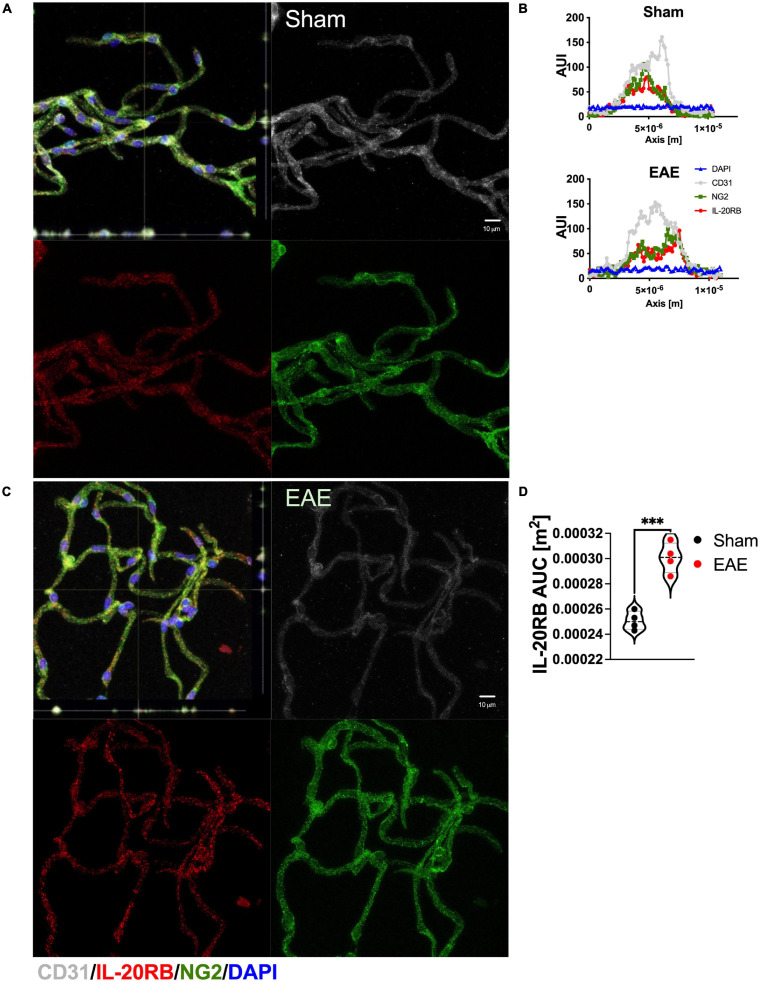
Enhanced expression of IL-20RB in C57BL/6 mice in spinal microvessels at peak of EAE. Spinal cord microvessels were isolated from sham-immunized and actively immunized C57BL/6 mice at peak of EAE (peak, ∼14 dpi), *n* = 4. Microvessels were immunolabeled for CD31 (white, endothelial marker), IL-20RB (red), and NG2 (green, mural/pericyte marker), and nuclear stained with DAPI (blue); scale bar = 10 μm **(A,C)**. IL-20RB colocalized with both CD31 and NG2, which suggests endothelial and mural cells expression of IL-20RB. Histogram analysis was performed to determine microvessels diameter to confirm low-caliber (not shown) and fluorescence arbitrary units of intensity (AUI) (representative AUI vs. axis plots, **B**). Area-under-curve (AUC) was calculated for IL-20RB AUI for 25 microvessels per subject. Means AUC for spinal cord IL-20RB expression of sham-immunized mice vs. actively immunized mice (mean ± SD: 2.5 × 10^–4^ ± 7.4 × 10^–6^ vs. 3.0 × 10^–4^ ± 1.6 × 10^–5^) were analyzed by unpaired *t*-test showing extreme statistically significant difference, *p* < 0.0001 **(D)**. Results are shown as mean ± SD, ***p < 0.001.

Likewise, we investigated expression of IL-20RB by isolating CNS region-specific microvessels from C57BL/6J mice, *n* = 4, sham-immunized and actively immunized for EAE (∼14 dpi, Figure 4). Expression of IL-20RB (red) was detected in spinal cord microvessels together with canonical cell markers to endothelial cells and mural cells, CD31 (white) and NG2, respectively (Figure 4A, representative images). Then, we analyzed 25 immunolabeled microvessels per mouse as previously described (Dayton et al., 2019; Yuan et al., 2020) for arbitrary-unit-of intensity (AUI, Figure 4B, representative histograms) to determine area-under-curve (AUC) which is proportionate to protein expression. Unpaired *t*-test of the mean differences between sham and EAE showed to be significant, *p* < 0.001 (IL-20RB AUC sham vs. EAE mean ± SD 2.5 × 10^–4^ ± 7.4 × 10^–6^ vs. 3.1 × 10^–4^ ± 1.2 × 10^–5^, Figure 4C).

In summary, these data demonstrate that both the IL-20 subfamily cytokines and IL-20 receptor subunit IL-20RB are present in the CNS of C57BL/6 mice. Moreover, that distinctive changes in protein expression levels occurred during EAE that may impact EAE neuropathology.

### Non-human Primate CNS Microvessels Expression of IL-20RB Is Region-Specific and IL-22RA1 Exhibits More Robust Expression in Human PVWM Microvessels

**FIGURE 5:**
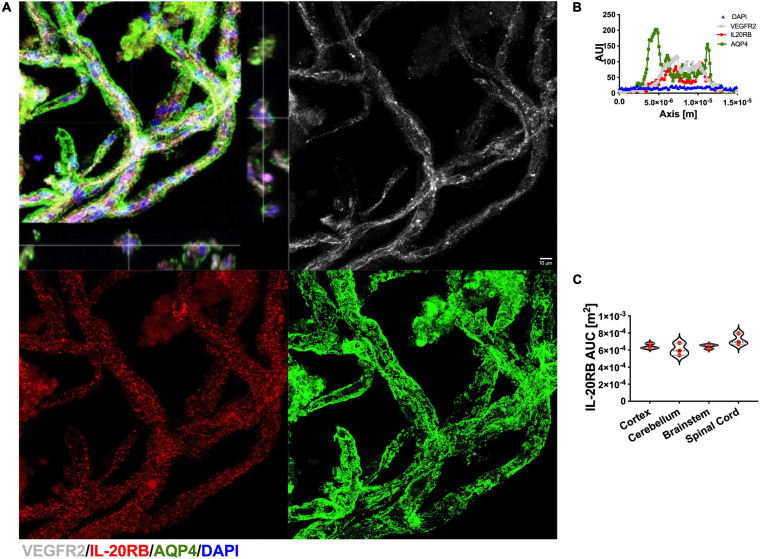
IL-20 receptor subunit β expression on rhesus macaque CNS microvessels. CNS region-specific microvessels were isolated from macaque tissue, *n* = 3, to elucidate IL-20RB protein expression by immunolabeling for VEGFR-2 (white, endothelial marker), IL-20RB (red), AQP4 (green, astrocytic end-feet marker), and DAPI (blue), scale bar = 10 μm **(A)**. VEGFR-2 colocalized with IL-20RB confirming endothelial cells expression of IL-20RB whereas AQP4 appears to envelop both. Histogram analysis was performed to 50 microvessels per specimen and CNS region to determine microvessels diameter and AUI for IL-20RB expression (representative AUI vs. axis plot, **B**). One-way ANOVA analysis followed by Sidak’s *post hoc* test showed no statistical significance between AUC means for the different CNS regions (mean ± SD cortex: 6.4 × 10^–4^ ± 2.9 × 10^–5^, cerebellum: 6.0 × 10^–4^ ± 7.3 × 10^–5^, brainstem: 6.4 × 10^–4^ ± 2.5 × 10^–5^ and spinal cord: 7.1 × 10^–4^ ± 6.7 × 10^–5^, **C**). Results are shown as mean ± SD.

Since, the possibility of performing isolation of human microvessels from multiple CNS regions presented a major limitation, we decided to perform this assay on a translational specie, rhesus macaque, as proof of concept. We isolated cortical, cerebellar, brainstem, and spinal microvessels from macaque tissues, *n* = 3, as previously described (Dayton et al., 2019; Yuan et al., 2020). Then, microvessels were immunolabeled with IL-20RB (red) antibody, VEGFR-2 (white) to label endothelial cells and AQP4 (green) to label astrocytic end-feet (Figure 5). We observed that IL-20RB colocalized with VEGFR-2 (white, Figure 5A) suggesting endothelial cell expression. Additionally, astrocytic end-feet marker AQP4 (green) clearly surrounded these lumen-forming cells, evidencing they conform the NVU. Quantification of AUI for IL-20RB (red) of 50 individual microvessels per sample from cortical, cerebellar, brainstem, and spinal microvessels immunolabeled was performed as described above for murine microvessels for each CNS region under investigation (representative histogram, Figure 5B). One-way ANOVA followed by Sidak’s *post hoc* test showed no significant differences among them (IL-20RB AUC mean ± SD: cortical 6.42 × 10^–4^ ± 2.95 × 10^–5^, cerebellar 6.04 × 10^–4^ ± 7.33 × 10^–5^, brainstem 6.42 × 10^–4^ ± 2.46 × 10^–5^, and spinal cord 7.19 × 10^–4^ ± 6.67 × 10^–5^, Figure 5C).

**FIGURE 6:**
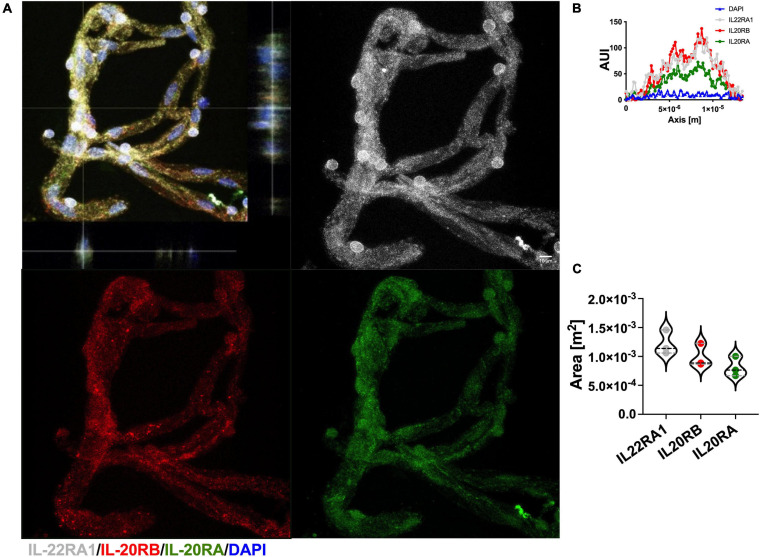
Human CNS microvasculature expresses IL-20 receptor subunits. Human microvessels were isolated from PVWM post-mortem biopsies, *n* = 3, to elucidate IL-20 receptor subunits protein expression by immunolabeling for IL-22RA1 (white), IL-20RB (red), IL-20RA (green), and DAPI (blue), scale bar = 10 μm **(A)**. Histogram analysis was performed to 50 microvessels per specimen to determine AUI and AUC of each receptor subunit (representative AUI vs. axis plot histogram, **B**). Although AUC means exhibit a trend IL-22RA1 > IL-20RB > IL-20RA, one-way ANOVA analysis followed by Sidak’s *post hoc* test showed that mean AUC differences between subunits are not statistically significant (mean ± SD IL-22RA1: 1.2 × 10^–3^ ± 2.1 × 10^–4^, IL-20RB: 9.9 × 10^–4^ ± 2.0 × 10^–4^, and IL-20RA: 8.1 × 10^–4^ ± 1.7 × 10^–4^, **C**). Results are shown as mean ± SD.

Recently, we obtained access to human post-mortem brain biopsies, in addition to reliable antibodies for IL-22RA1 and IL-20RA immunolabeling. Because of this, we were able to isolate microvessels from PVWM, *n* = 3, to elucidate IL-20 receptor subunits protein expression using IL-22RA1 (white), IL-20RB (red), and IL-20RA (green) antibodies (Figure 6A, representative image). We proceed as afore mentioned to further quantitate protein expression by histogram analyses of 50 individual microvessels per sample, to determine AUC for each IL-20 receptor subunit (representative AUI vs. axis plot histogram, Figure 6B). We analyzed by one-way ANOVA followed by Sidak’s *post hoc* test and observed a trend in expression level for IL-22RA1 > IL-20RB > IL-20RA but no statistical significance (IL-22RA1 AUC mean ± SD 1.22 × 10^–3^ ± 2.11 × 10^–4^, IL-20RB AUC mean ± SD 9.94 × 10^–4^ ± 2.01 × 10^–4^, and IL-20RA mean ± SD 8.10 × 10^–4^ ± 1.72 × 10^–4^, Figure 6C). A similar expression pattern was observed for microvessels isolated from naïve C57BL/6J mice, *n* = 3, CNS tissue (Supplementary Figure 7). The expression of all IL-20 receptor subunits within human PVWM microvessels strongly suggest IL-20 subfamily cytokines to be prevalent within this CNS region with known involvement in MS neuropathology.

### A Pro-inflammatory Condition Induces Augmentation of IL-20 Receptor Subunits Expression in HCMEC/D3 Cells and IL-20 Cytokine Stimulates Alteration of CXCL12 Polarity in Absence of Other Pro-inflammatory Mediators

**FIGURE 7:**
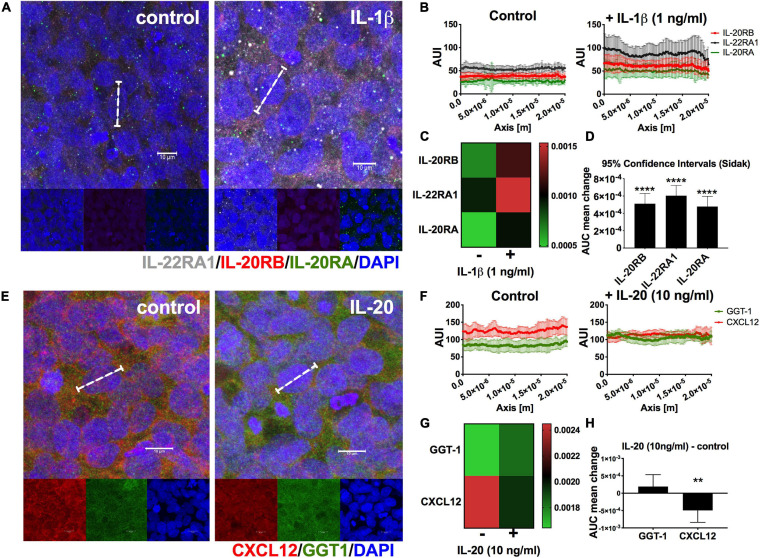
Expression of IL-20 receptors and IL-20-mediated distortion in CXCL12 polarity in HCMEC/D3 cells. HCMEC/D3 seeded on eight-well slides were incubated for 24 h in the presence or absence of 1 ng/ml rhIL-1β, *n* = 3. After 24 h, cells were immunolabeled for IL-22RA1 (white), IL-20RB (red), and IL-20RA (green) using DAPI (blue) for nuclear staining **(A)**. A total of 50 cells per group were analyzed to determine AUC as estimate for receptor subunits expression levels (dashed bracket shown a representative single cell, scale bar = 10 μm) **(A)**. Graphic summary of AUI for control or rhIL-1β-treated HCMEC/D3 cells, *n* = 50, used to calculate AUC **(B)**. Heatmap of change in AUC distribution of IL-20RA, IL-20RB, and IL-22RA1 between control and rhIL-1β **(C)**. Two-way ANOVA followed by Sidak’s *post hoc* test showed that mean differences are statistically significant for all receptor subunits. Results are shown as mean ± SD, *p* < 0.0001. A similar approach was used to elucidate if IL-20 exerted changes in CXCL12 apicobasal polarity **(D)**. HCMEC/D3 were treated with 10 ng/ml rhIL-20, *n* = 3, for 24 h, and immunolabeled for basal marker CXCL12 (red), apical marker GGT1 (green), and DAPI (blue) for nuclear staining **(E)**. White dashed bracket shows representative single cell used for histogram analysis, scale bar = 10 μm **(E)**. Graphic summary of AUI used to determine AUC, *n* = 25, for control or rhIL-20-treated HCMEC/D3 cells **(F)**. Heatmap of mean change in AUC distribution of GGT1 and CXCL12 between control and IL-20-treated cells **(G)**. Two-way ANOVA followed by Sidak’s *post hoc* test, showed a statistically significant change for CXCL12 and not for GGT1 **(H)**. Results are shown as mean ± SD, **p < 0.01 and ****p < 0.0001.

To elucidate if IL-20 subfamily cytokines signaling play a role in human NVU and potential triggers of BBB permeability and apicobasal polarity modulators, we turned to the HCMEC/D3 cells (Daniels et al., 2013; Cruz-Orengo et al., 2014). First, we wanted to identify if HCMEC/D3 cells constitutively expressed IL-20 receptor subunits by immunocytochemistry (ICC) and if such expression is susceptible to pro-inflammatory mediators like IL-1β (Figure 7A). We labeled HCMEC/D3 cells with antibodies toward IL-22RA1 (white), IL-20RB (red), and IL-20RA (green) with and without stimulating the cells with 1 ng/ml of rhIL-1β for 24 h. We observed expression of all IL-20 receptor subunits (Figure 7A). Moreover, we quantitated the distribution of protein expression within individual cells, as assessed by histogram analysis, and found an increase in protein distribution between rhIL-1β-treated cells and control (Figures 7B,C). Two-way ANOVA followed by Sidak’s *post hoc* test of the AUI mean ± SD showed differences to be extremely statistically significant, *p* < 0.0001, for the three receptor subunits (Figure 7C, heatmap; Figure 7D, 95% confidence interval). It also found that rhIL-1β cytokine treatment accounts for a 45.4% of the total variance. These data confirm the IL-22RA1, IL-20RB, and IL-20RA protein expression in human brain microvascular endothelium and that pro-inflammatory stimuli could intensify such expression. Furthermore, it suggests that IL-20 subfamily cytokines could signal the NVU endothelium by either Type 1 or Type 2 IL-20 receptor.

Knowing that IL-20 receptor subunits are expressed in HCMEC/D3 cells, we decided to elucidate if they are capable of IL-20 signaling and if this stimulation could exert changes relevant to neuroinflammation. We stimulated HCMEC/D3 cells for 24 for hours in rhIL-20, 10 ng/ml and performed ICC against CXCL12 (red), known as molecular gate keeper for immune extravasation. Since, CXCL12 is normally expressed at the basal surface of the NVU endothelium basal surface, we also used GGT1 (green) as canonical marker of NVU-endothelium apical surface, to accurately assess CXCL12 re-distribution. GGT1 is a glycosylated protein embedded in the apical of the plasma membrane responsible for transferring glutamyl groups and maintaining glutathione and cysteine homeostasis (Neuman et al., 2020). HCMEC/D3 cells showed GGT1 and CXCL12 expression as anticipated (Figure 7E). We quantitated GGT1 and CXCL12 expression (Figure 7F) to determine if the presence of rhIL-20 could alter any of the polarity of these proteins. Two-way ANOVA of mean ± SD followed by Sidak’s *post hoc* showed a statistically significant change for CXCL12 distribution and not for GGT1, *p* < 0.01 (Figures 7G,H, heatmap and 95% confidence interval for GGT1 and CXCL12 changes in distribution, respectively). These data support that IL-20 signaling could modulate NVU apicobasal polarity. Moreover, it stresses that IL-20 preferentially targets CXCL12 redistribution, facilitating immune extravasation.

## Discussion

Since the discovery of the IL-20 subfamily of cytokines and receptors, there has been a growing interest in understanding their role in inflammation, particularly cancer, tissue repair, and autoimmunity (Ouyang et al., 2011; Rutz et al., 2014). Members of the larger IL-10 family, they had been grouped as a subfamily based on their usage of the same receptor subunits, the biological functions they exert and their cellular targets (Ouyang et al., 2011; Rutz et al., 2014). Ever since Rutz et al. review on IL-20 subfamily new cell targets had been identified, including astrocytes (Cooley et al., 2014; Rutz et al., 2014; Burmeister and Marriott, 2018; Burmeister et al., 2019a,b). Moreover, astrocytes also produce IL-19, another member of the IL-20 subfamily, which suggests an autocrine mechanism to IL-20 pro-inflammatory signaling (Cooley et al., 2014; Burmeister and Marriott, 2018; Burmeister et al., 2019a). Additionally, astrocytes are reported to secrete VEGF-A, and consequently modulate angiogenesis and endothelial cells barrier properties (Krum and Rosenstein, 1998; Krum and Khaibullina, 2003; Croll et al., 2004; Argaw et al., 2006, 2009, 2012; Suidan et al., 2010; Chapouly et al., 2015). On the other hand, IL-20 promotes VEGF-mediated angiogenesis including other neuroinflammatory and autoimmune pathology: e.g., myasthenia gravis (MG) (Hsieh et al., 2006; Hammer et al., 2009; Baird et al., 2011; Aydogdu et al., 2013; Alexandrakis et al., 2015; Han et al., 2016; Kako et al., 2016; Uzawa et al., 2016; Huang et al., 2018). Although MG is an autoimmune pathology that targets the peripheral nervous system and not the CNS, it is noteworthy that a research group reported a significant increase in IL-19 and IL-20 serum levels similar to other well characterized pro-inflammatory cytokines that impact CNS (Uzawa et al., 2016). The same study found a significant decrease in IL-19 and IL-20 serum levels in patients after immunosuppressive therapy, suggesting these could be used as indicators of treatment responsiveness (Uzawa et al., 2016).

Before this backdrop, we initiated our investigation on the role of IL-20 and IL-20RB signaling at the BBB using EAE, murine model of MS, and well characterized model of neuroinflammation and neuropathology. Our initial attempt was to elucidate if genetic ablation of IL-20 has any impact on EAE pathogenesis. Unfortunately, *IL-20^–/–^* were embryonic lethal and we decided to use *IL-20^+/–^* instead, which didn’t yield the expected outcome of significantly lower disease severity (Supplementary Figure 2). We then turned our attention to *IL-20RB^–/–^* mice. Our rationale behind this approach is first because there are no available knock out for either IL-20RA or IL-22RA1. Secondly, because IL-20RB subunit is required for both IL-20 receptors, Type 1 and Type 2, thus targeting IL-20RB seemed as the best approach to answer this inquiry. Indeed, we did observe noticeable changes on EAE neuropathology with genetic ablation of IL-20RB subunit. Although *IL-20RB^–/–^* mice actively immunized for EAE did develop EAE, clinical score was not statistically different to sham-immunized *IL-20RB^–/–^* mice (Figure 1A, white circle and red line), opposite to the comparison with actively immunized wild-type mice (Figure 1A, black circle and line). Also, actively immunized *IL-20RB^–/–^* mice maintained their body weight similar way to sham-immunized *IL-20RB^–/–^* mice and not to wild-type mice with EAE (Figure 1A). The same could be said for the other metrics of disease severity: highest and cumulative scores, and day of disease onset (Figures 1B–D). All these metrics demonstrate that *IL-20RB^–/–^* mice fare better than wild-type mice during EAE. Since EAE neuropathology is predominantly observed at the spinal cord, we performed histology to *IL-20RB^–/–^* mice and wild-type mice at peak of disease severity, ∼14 dpi (Supplementary Figure 1). Consistent with other studies using H&E to assess neuroinflammation (Cruz-Orengo et al., 2011, 2014), *IL-20RB^–/–^* mice presented less perivascular infiltrates at spinal cord white matter than wild-type mice as expected for their clinical score (Supplementary Figure 1 and Figure 1A).

However, our strongest *in vivo* finding supporting that IL-20 receptor subunits, particularly IL-20RB, are relevant for EAE neuropathology comes from the adoptive transfer experiments (Figure 2). Overall, these data clearly demonstrate that IL-20RB expression at the CNS does play a relevant role in EAE neuropathology. As detailed described in the Results section, the differences between wild-type mice host and *IL-20RB^–/–^* mice host are salient (Figure 2). Remarkably, *IL-20RB^–/–^* → wild-type mice clinical score skewed toward the left (Figure 2A, red circle with black outline). This could be interpreted as lack of IL-20RB within the immune system resulting in more reactivity upon MOG-Th1 cells. However, CD4+ cells do not express IL-20RB (Ouyang et al., 2011; Rutz et al., 2014). Additionally, this skewness could be associated with an early disease onset (Figure 2D, *p* < 0.05). Although the adoptive transfer was conducted with MOG-Th1 cells and the active immunization model is highly skewed toward a Th1 cell repertoire, we can’t ignore that many other immune cells contribute to EAE neuroinflammation. Peripheral myeloid cells, including macrophages, dendritic cells, and neutrophils, exhibit a prominent role during EAE, and are a major component of MS lesions (Levesque et al., 2016; Giles et al., 2018; Tsai et al., 2019; Ifergan and Miller, 2020; Lu et al., 2020; Melero-Jerez et al., 2020; Owens et al., 2020; Tanwar et al., 2020; Wasser et al., 2020). Likewise, myeloid cells are both primary sources and targets of IL-20 subfamily cytokines (Hsu et al., 2006, 2011; Kragstrup et al., 2008; Wolk et al., 2008, 2009a; Wang et al., 2012; Rutz et al., 2014; Mayer et al., 2015; Bech et al., 2016; Kako et al., 2016; Gough et al., 2017; Senolt et al., 2017; Zhang et al., 2017; Dabitao et al., 2018; Niess et al., 2018; Weng et al., 2019). That said, *IL-20RB^–/–^* host mice are deficient of IL-20 subfamily cytokines signaling on both nitches, CNS and peripheral myeloid cells, thus EAE amelioration exhibited by these mice is also due to a deficit in dendritic cell maturation, neutrophil infiltration, and diminished expression of IL-6, IFN-γ, and TNF-α by monocytes and PBMC (Hsu et al., 2006, 2017b; Kragstrup et al., 2008, 2017; Hsu and Chang, 2010; Baird et al., 2011; Rutz et al., 2014; Alexandrakis et al., 2015; Hofmann et al., 2015; Mayer et al., 2015; Kako et al., 2016; Chen et al., 2018; Dabitao et al., 2018). Future studies in our laboratory will further distinguish the specific contributions of both nitches, CNS (astrocytes, microglia, oligodendrocytes, and NVU) and peripheral myeloid cells in this context. Still, to our knowledge, this is the first study directly linking IL-20 subfamily with EAE neuropathology. This opens the gate to a putative association with neuroinflammatory disorders such as MS.

Unfortunately, neutralization of IL-20 with MABIL-20 treatment during EAE didn’t provide a favorable outcome (Supplementary Figure 4). A plausible interpretation is that MABIL-20 treatment needed to be extended for more than 10 days since MABIL-20-treated EAE showed a rebound in clinical score with treatment interruption (Supplementary Figure 4A). However, we performed statistical analysis of highest and cumulative scores comparing treatment and post-treatment periods and were deemed not significant (data not shown). Another possibility is that treatment needed to be initiated prior to disease onset. Our reasoning behind initiating treatment with a score of 2, was based on the consideration that treatment for human neurologic disorders is commonly prescribed after diagnosing, not prophylactic. It can also be explained by the fact that IL-19 and IL-24 are also present in murine CNS and neutralizing only IL-20 is not enough to provoke a therapeutic benefit (Figure 3; Cooley et al., 2014; Burmeister et al., 2019a).

Consistent with the Burmeister et al. reports (Cooley et al., 2014; Burmeister et al., 2019a) regarding astrocytic expression of IL-20 subfamily, we did find IL-19, IL-20, and IL-24 in murine CNS tissues by ELISA (Figure 3 and Supplementary Figures 5–7). Moreover, decrease in many relevant cytokine expression levels at the spinal cord were robust for *IL-20RB^–/–^* mice during EAE compared to wild-type mice (Figure 3), including IL-20 and IL-24 (Figures 3O,P). This is relevant since EAE exhibits an ascending paralysis disease progression and neuropathology and neuroinflammation are more preponderant at the spinal cord. As aforementioned, astrocytes and microglia are sources and targets of IL-20 subfamily cytokines, and many immune cells are known to produce IL-20 subfamily cytokines: T and B cells, monocytes, macrophages, granulocytes, dendritic cells, etc. (Cooley et al., 2014; Rutz et al., 2014; Burmeister and Marriott, 2018; Chen et al., 2018; Burmeister et al., 2019a,b). That said, we are currently pursuing the sources of IL-20 subfamily cytokines responsible for the expression levels observed for during EAE.

IL-20 receptor subunit β expression at spinal cord microvasculature heightened during EAE (Figure 4). We are confident of this finding, since this method was specifically designed to harvest microvessels from CNS parenchyma, eliminating high-caliber vessels (Dayton et al., 2019; Yuan et al., 2020). Consistent with our hypothesis, IL-20RB expression colocalized with CD31, underlining endothelial cells contribution to IL-20RB expression in CNS microvasculature (Figures 4A–C). However, mural cells seem to also express IL-20RB as suggested by the colocalization with NG2 (Figures 4A–C). At the time we initiated this project, the CNS region-specific microvessels isolation method was only validated for fresh CNS tissues. To solve this limitation, we turned to rhesus macaque as proof-of-concept. As anticipated, IL-20RB colocalized with endothelial marker VEGF-R2 (Figure 5A). Although the results reported are from macaques without neurologic or neuroinflammatory conditions, they are still relevant because they open the possibility of translational studies with the non-human primate (Figure 5).

Presently, we moved forward with the use of human post-mortem brain biopsies. We decided to initiate our inquiry using PVWM from young female (31 and 32 years-old) individuals, *n* = 3. The reasoning behind this choice is multiple. First, EAE is the murine model for MS thus it is reasonable to initiate our inquiry building data toward research focusing on MS neuropathology. Secondly, MS is a sex-biased neurologic disorder affecting 3.5 more women than men, and the PVWM exhibits a preponderance of active MS lesions in most patients. Third, we wanted to avoid confounding neuroinflammatory effects related to aging. In addition to our newly access to human CNS tissues, validated IL-22RA1 and IL-20RA antibodies are now also available. We were able to identify and quantitate expression of IL-20 receptor subunits in the microvessels isolated from the PVWM samples (Figure 6). Furthermore, we were able to observe a distinctive trend in expression levels for each subunit behind IL-22RA1 the highest and IL-20RA the lowest. We also observed the same trend in naïve murine microvessels (Supplementary Figure 9). This is relevant because, IL-20 receptor Type 2 is formed by dimerization of IL-22RA1 and IL-20RB subunits thus these data support that Type 2 may be the dominant receptor. Although this view warrants further investigation, to our knowledge this is the first report of IL-20 receptor subunits expression in human CNS microvasculature and within a region with known relevance to human neuropathology, particularly MS.

Following this line of thought, we also found that HCMEC/D3 cells commonly used to model human BBB, also expressed all IL-20 receptor subunits (Figure 7A). This is relevant because although it was known that endothelial cells are target to IL-20 cytokine (Rutz et al., 2014; Chen et al., 2018), the expression of IL-20 receptor subunits specifically within microvessels associated with BBB function was unidentified. In harmony with the data obtained with human PVWM microvessels, expression levels of these subunits showed the same IL-22RA1 > IL-20RB > IL-20RA trend in expression pattern (Figures 7A,B). We also unveiled the impact of pro-inflammatory triggers in the expression of IL-20 receptor subunits as observed by the augmentation of protein expression with rhIL-1β treatment (Figures 7A–D). This is significant first, because supports that signaling of IL-20 subfamily cytokines occurs during neuroinflammatory processes. Secondly, IL-1β is known to cause BBB dysfunction and IL-20 subfamily cytokines may be enhancing IL-1β potency in a synergistic fashion (Argaw et al., 2006). Although augmentation with rhIL-1β treatment showed extreme statistical significance for all IL-20 receptor subunits compare to control, IL-22RA1 showed a more pronounce effect (Figures 7A–D).

Concordant with our main hypothesis, we demonstrated that HCMEC/D3 cells are susceptible to rhIL-20 treatment, mediating a distinctive alteration to CXCL12 apicobasal polarity (Figures 7E–H). This is relevant because CXCL12 redistribution to apical, a.k.a. luminal, endothelial cell surfaces facilitates lymphocyte extravasation during neuroinflammation (Cruz-Orengo et al., 2011, 2014; Daniels et al., 2013, 2014). This role as BBB “immune-gate-keeper” had been reported in EAE, MS, and neurotropic viral infection, *in vivo* and using HCMEC/D3 cells *in vitro* (Cruz-Orengo et al., 2011, 2014; Daniels et al., 2013, 2014). In addition GGT1, a known marker of BBB function but not involved in immune extravasation, didn’t show the same redistribution (Kuchler-Bopp et al., 1999; Orte et al., 1999; Hosoya et al., 2000; Kaiser et al., 2006; Yu et al., 2007; Vandenhaute et al., 2011). Although GGT1 is better characterized for its role in protein synthesis, transmembrane transport, and glutathione/cysteine homeostasis, its role in triggering brain-barrier specific properties to endothelial cells had also been noted thus heightening the relevance of CXCL12 redistribution (Kuchler-Bopp et al., 1999; Orte et al., 1999; Hosoya et al., 2000; Cornford and Hyman, 2005; Yu et al., 2007; Vandenhaute et al., 2011; Kunutsor et al., 2014, 2015; Neuman et al., 2020).

Vascular endothelial growth factor-A production and VEGFR-2 expression had been reported on HCMEC/D3 cells and their modulation in the presence of pro-inflammatory cytokines (TNFα, TGFβ) and other conditions (hypoxia, altered glycemia, etc.) (Yang et al., 2013; Sajja et al., 2014; Jacob et al., 2015; Krishnan et al., 2015; Yang and Friedl, 2015; Chen et al., 2019; Zhang et al., 2019). Also, HCMEC/D3 cells had been reported to exhibit counter-modulatory, or so called “Yin-Yang,” effects like the one observed with sphingosine-1-phosphate receptors S1PR1 and S1PR2 (Cruz-Orengo et al., 2014). Presumably the same may be occurring with IL-20 subfamily cytokine signaling. Since subunits for both, Type 1 and Type 2, are present it is important to characterize the triggering factors that may swift to a preponderance of Type 1 or Type 2 by either upregulation or downregulation of either IL-22RA1 or IL-20RA subunits. These factors could be a sudden increase in IL-19 levels within the CNS, increase levels of pro-inflammatory or anti-inflammatory cytokines, or other mechanisms as reported for NSCLC, HUVEC, HMEC, and aortic rings (Hsieh et al., 2006; Hammer et al., 2009; Baird et al., 2011; Kako et al., 2016). Although angiogenic effects had been reported only with IL-19 and IL-20, it is intriguing to consider that IL-24 might have a similar effect but specifically within the CNS. Likewise, IL-20 receptors signaling may trigger VEGF-dependent activity, that could be either astrocyte-derived or autocrine (Hsieh et al., 2006; Argaw et al., 2009, 2012; Hammer et al., 2009; Davis et al., 2010; Baird et al., 2011; Chapouly et al., 2015; Han et al., 2016; Spampinato et al., 2017). Further studies in our laboratory are trying to elucidate this possibility and the mechanisms downstream signaling of IL-20 subfamily cytokines, like phosphorylation of ERK1/2, p38, JNK, Erk1/2, PI3K, and mTOR, and STAT3 activity for consequent transcription of VEGF, VEGFR-2 activity, and immune extravasation (Hsieh et al., 2006; Argaw et al., 2009, 2012; Hammer et al., 2009; Davis et al., 2010; Suidan et al., 2010, 2012; Baird et al., 2011; Rodrigues and Granger, 2015; Kako et al., 2016; Suzuki et al., 2016; Spampinato et al., 2017; Michinaga and Koyama, 2019).

Finally, this study shows evidence of a pivotal role for IL-20 subfamily cytokines signaling during EAE, a canonical mouse model of neuroinflammation and neuropathology. Based on the data presented, we propose that IL-20 receptor subunits expression by NVU endothelial cells may be responsible for immune extravasation during EAE by distorting CXCL12 apicobasal polarity at the microvasculature that conforms the BBB. More analysis is needed to determine the sources of IL-20 subfamily cytokines, within the CNS or peripheral cells or tissues, to further understand the molecular mechanisms by which IL-20 subfamily cytokines influences the development of EAE neuropathology. Moreover, we need to elucidate the mechanisms downstream of signaling of IL-20 subfamily cytokines that leads to CXCL12 redistribution and if they are dependent or independent of VEGF activity. Ultimately, our efforts are oriented toward proposing IL-20 subfamily cytokines as therapeutic target for neuroinflammatory pathologies, like MS, autism, Alzheimer’s disease, and post-stroke recovery.

## Data Availability Statement

The raw data supporting the conclusions of this article will be made available by the authors, without undue reservation.

## Ethics Statement

The animal study was reviewed and approved by UC Davis Institutional Animal Care and Use Committee protocol #22012.

## Author Contributions

JD contributed with experiment design and execution, data collection, data analysis, and manuscript editing. YY contributed with experiment execution, data collection, and data analysis. LP, BD, SY, MO, and MM contributed with experiment execution and data collection. SH-S contributed with data analysis and manuscript editing. LC-O contributed with experiment design and execution, data collection, data analysis, and manuscript writing. All authors contributed to the article and approved the submitted version.

## Conflict of Interest

The authors declare that the research was conducted in the absence of any commercial or financial relationships that could be construed as a potential conflict of interest.

## Publisher’s Note

All claims expressed in this article are solely those of the authors and do not necessarily represent those of their affiliated organizations, or those of the publisher, the editors and the reviewers. Any product that may be evaluated in this article, or claim that may be made by its manufacturer, is not guaranteed or endorsed by the publisher.

## Supplementary Material

Supplementary Figure 1 *IL-20RB^–/–^* mice exhibits reduction of perivascular infiltrates. *IL-20RB^–/–^* mice and wild-type mice were actively immunized for EAE and euthanized at peak (∼14 dpi) for histological analysis, *n* = 4. H&E staining was performed on spinal cords, following standardized protocols (representative image, scale bar = 100 μm, **A**). Means of total number of perivascular infiltrates throughout ventral white matter were analyzed by unpaired *t*-test showed statistical significance between wild-type and *IL-20RB^–/–^* mice (mean ± SEM: 560.4 ± 41.15 vs. 198.4 ± 63.06) *p* < 0.01 **(B)**. Perivascular infiltrates were also analyzed based on location: ventromedial white matter, VMWM; ventral column white matter, VCWM, and ventrolateral white matter, VLWM **(C)**. Comparison of perivascular infiltrates between wild-type and *IL-20RB^–/–^* mice and analysis by two-way ANOVA analysis followed by Sidak’s *post hoc* test showed statistical significance for VMWM (mean ± SEM: 210.75 ± 20.90.15 vs. 57.75 ± 18.96), *p* < 0.01, VCWM (mean ± SEM: 239.63 ± 24.96.15 vs. 83.00 ± 25.81), and VLWM (mean ± SEM: 140.88 ± 24.60 vs. 57.63 ± 18.34), *p* < 0.05 **(D)**. Results are shown as mean ± SEM, ^∗^*p* < 0.05 and ^∗∗^*p* < 0.01.

Supplementary Figure 2 Heterozygosity of IL-20 does not alter EAE disease progression. Ten week-old *IL-20^–/–^* mice, male and female, were actively immunized for EAE (red circle) or sham-immunized (black circle) as control (*n* = 10). Clinical score, body weight, highest and cumulative scores, from 7 to 28 dpi were analyzed by unpaired *t*-test **(A–C)**. Difference in clinical score and body weight, highest and cumulative scores, between actively immunized *IL-20^+/–^* mice and control showed extreme statistical significance, *p* < 0.0001 (mean ± SEM clinical score: 1.14 ± 0.18 vs. 0.00 ± 0.00, body weight: 20.59 ± 0.14 vs. 22.28 ± 0.15, **A**) (mean ± SEM highest score: 2.95 ± 0.38 vs. 0.00 ± 0.00, **B**) (mean ± SEM cumulative score: 22.50 ± 3.41 vs. 0.00 ± 0.00, **C**). Disease onset of actively immunized *IL-20^+/–^* mice with neurologic symptoms (10/10, mean ± SEM: 14.20 ± 0.42) **(D)**. Analysis of percentage of survival was not deemed significant **(E)**. Results are shown as mean ± SEM, ^****^*p* < 0.0001.

Supplementary Figure 3 Necropsy of sham-immunized mice treated with MABIL-20 and IgG_2__*B*_. C57BL6/J mice, male and female, *n* = 4, were treated with 10 mg/kg BW of rat monoclonal anti-IL-20 (MABIL-20) and IgG_2B_ isotype control, 10 days after sham immunization to assess safe use of monoclonal antibody. Treatment continued for 10 days, then mice were euthanized for histological analysis. Comparison between MABIL-20 and IgG_2B_ tissues were unremarkable. Representative images for brain **(A)**, skin **(B)**, lung **(C)**, knee **(D)**, spleen **(E)**, cervical, axillary, inguinal lymph nodes (**F–H**, respectively), kidney **(I)**, liver **(J)**, stomach **(K)**, small and large intestine (**L,M**, respectively).

Supplementary Figure 4 Neutralization of IL-20 cytokine has no impact on clinical progression of EAE. Ten week-old C57BL6/J mice, male and female, were actively immunized for EAE, sham-immunized (not show) as control (EAE, *n* = 12; sham, *n* = 4). Treatment with 10 mg/kg BW of rat monoclonal anti-IL-20 (MABIL-20) and IgG_2B_ isotype control started when mice reached a score or 2 (hindlimb paresis). Clinical score, body weight, highest and cumulative scores from 7 to 35 dpi were analyzed by unpaired *t*-test and changes were not deemed significant **(A–C)**. Analysis of survival curve was also not significant **(D)**. Results are shown as mean ± SEM.

Supplementary Figure 5 Cortical cytokine expression of wild-type mice and *IL-20RB^–/–^* mice at peak of EAE. Cytokines and VEGF expression within cortical tissues were detected by multianalyte bead-based immunoassay **(A–M)** and ELISA **(N–P)**, *n* = 4 (same cohort of mice shown on Figure 3 and Supplementary Figures 6–8), for wild-type mice at peak of EAE (peak, ∼14 dpi) (black circle) and sham-immunized, *IL-20RB^–/–^* mice (red circle), and sham-immunized (sham-immunized not shown). One-way ANOVA analysis followed by Sidak’s *post hoc* comparing wild-type mice and *IL-20RB^–/–^* mice at peak of EAE showed statistical significance for IL-23, *p* < 0.05 **(H)**, GM-CSF, *p* < 0.01 **(J)**, TNF-α, *p* < 0.05 **(L)**, and IL-24, *p* < 0.0001 **(P)**. Results are shown as mean ± SEM, ^∗^*p* < 0.05, ^∗∗^*p* < 0.01, and ^****^*p* < 0.0001.

Supplementary Figure 6 Cerebellar cytokine expression of wild-type mice and *IL-20RB^–/–^* mice at peak of EAE. Cytokines and VEGF expression within cerebellar tissues were detected by multianalyte bead-based immunoassay **(A–M)** and ELISA **(N–P)**, *n* = 4 (same cohort of mice shown on Figure 3 and Supplementary Figure 5), for wild-type mice at peak of EAE (peak, ∼14 dpi) (black circle) and sham-immunized, *IL-20RB^–/–^* mice (red circle), and sham-immunized (sham-immunized not shown). One-way ANOVA analysis followed by Sidak’s *post hoc* comparing wild-type mice and *IL-20RB^–/–^* mice at peak of EAE showed statistical significance only for GM-CSF, *p* < 0.05 **(J)** Results are shown as mean ± SEM, ^∗^*p* < 0.05.

Supplementary Figure 7 Cytokine expression at the brainstem of wild-type mice and *IL-20RB^–/–^* mice at peak of EAE. Cytokines and VEGF expression within brainstem tissues were detected by multianalyte bead-based immunoassay **(A–M)** and ELISA **(N–P)**, *n* = 4 (same cohort of mice shown on Figure 3 and Supplementary Figure 5), for wild-type mice at peak of EAE (peak, ∼14 dpi) (black circle) and sham-immunized, *IL-20RB^–/–^* mice (red circle), and sham-immunized (sham-immunized not shown). One-way ANOVA analysis followed by Sidak’s *post hoc* comparing wild-type mice and *IL-20RB^–/–^* mice at peak of EAE showed statistical significance for GM-CSF, *p* < 0.01 **(J)** and TNF-α, *p* < 0.001 **(L)**. Results are shown as mean ± SEM, ^∗^*p* < 0.05 and ^∗∗∗^*p* < 0.001.

Supplementary Figure 8 Cytokine expression from serum of wild-type mice and *IL-20RB^–/–^* mice at peak of EAE. Serum cytokines and VEGF levels were detected by multianalyte bead-based immunoassay (A-M), *n* = 4 (same cohort of mice shown on Figure 3 and Supplementary Figure 5), for wild-type mice at peak of EAE (peak, ∼14 dpi) (black circle) and sham-immunized, *IL-20RB^–/–^* mice (red circle), and sham-immunized (sham-immunized not shown). Data was analyzed by one-way ANOVA analysis followed by Sidak’s *post hoc* and changes were not deemed significant. Results are shown as mean ± SEM.

Supplementary Figure 9 Murine expression of IL-20 receptor subunits. Murine microvessels were isolated from naïve C57BL/6J mice, *n* = 3, to elucidate IL-20 receptor subunits protein expression by immunolabeling for IL-22RA1 (white), IL-20RB (red), IL-20RA (green), and DAPI (blue), scale bar = 10 μm **(A)**. Histogram analysis was performed to 25 microvessels per specimen to determine AUI and AUC of each receptor subunit (representative AUI vs. axis plot histogram, **B**). Although AUC means exhibit a trend IL-22RA1 > IL-20RB > IL-20RA, one-way ANOVA analysis followed by Sidak’s *post hoc* test showed that mean AUC differences between subunits are not statistically significant (mean ± SD IL-22RA1: 6.2 × 10^–4^ ± 2.2 × 10^–4^, IL-20RB: 4.2 × 10^–4^ ± 1.7 × 10^–4^, and IL-20RA: 5.4 × 10^–4^ ± 2.1 × 10^–4^, **C**). Results are shown as mean ± SD.
