# Supplementary material for: Expression of IL-20 Receptor Subunit β Is Linked to EAE Neuropathology and CNS Neuroinflammation
Source: Front Cell Neurosci. 2021 Sep 7;15:683687. doi: 10.3389/fncel.2021.683687 (PMC8452993; doi:10.3389/fncel.2021.683687)
Supplement: Supplementary Figure 1 — IL-20RB–/– mice exhibits reduction of perivascular infiltrates. IL-20RB–/– mice and wild-type mice were actively immunized for EAE and euthanized at peak (∼14 dpi) for histological analysis, n = 4. H&E staining was performed on spinal cords, following standardized protocols (representative image, scale bar = 100 μm, A). Means of total number of perivascular infiltrates throughout ventral white matter were analyzed by unpaired t-test showed statistical significance between wild-type and IL-20RB–/– mice (mean ± SEM: 560.4 ± 41.15 vs. 198.4 ± 63.06) p < 0.01 (B). Perivascular infiltrates were also analyzed based on location: ventromedial white matter, VMWM; ventral column white matter, VCWM, and ventrolateral white matter, VLWM (C). Comparison of perivascular infiltrates between wild-type and IL-20RB–/– mice and analysis by two-way ANOVA analysis followed by Sidak’s post hoc test showed statistical significance for VMWM (mean ± SEM: 210.75 ± 20.90.15 vs. 57.75 ± 18.96), p < 0.01, VCWM (mean ± SEM: 239.63 ± 24.96.15 vs. 83.00 ± 25.81), and VLWM (mean ± SEM: 140.88 ± 24.60 vs. 57.63 ± 18.34), p < 0.05 (D). Results are shown as mean ± SEM, ∗p < 0.05 and ∗∗p < 0.01. [file Image_1.pdf]

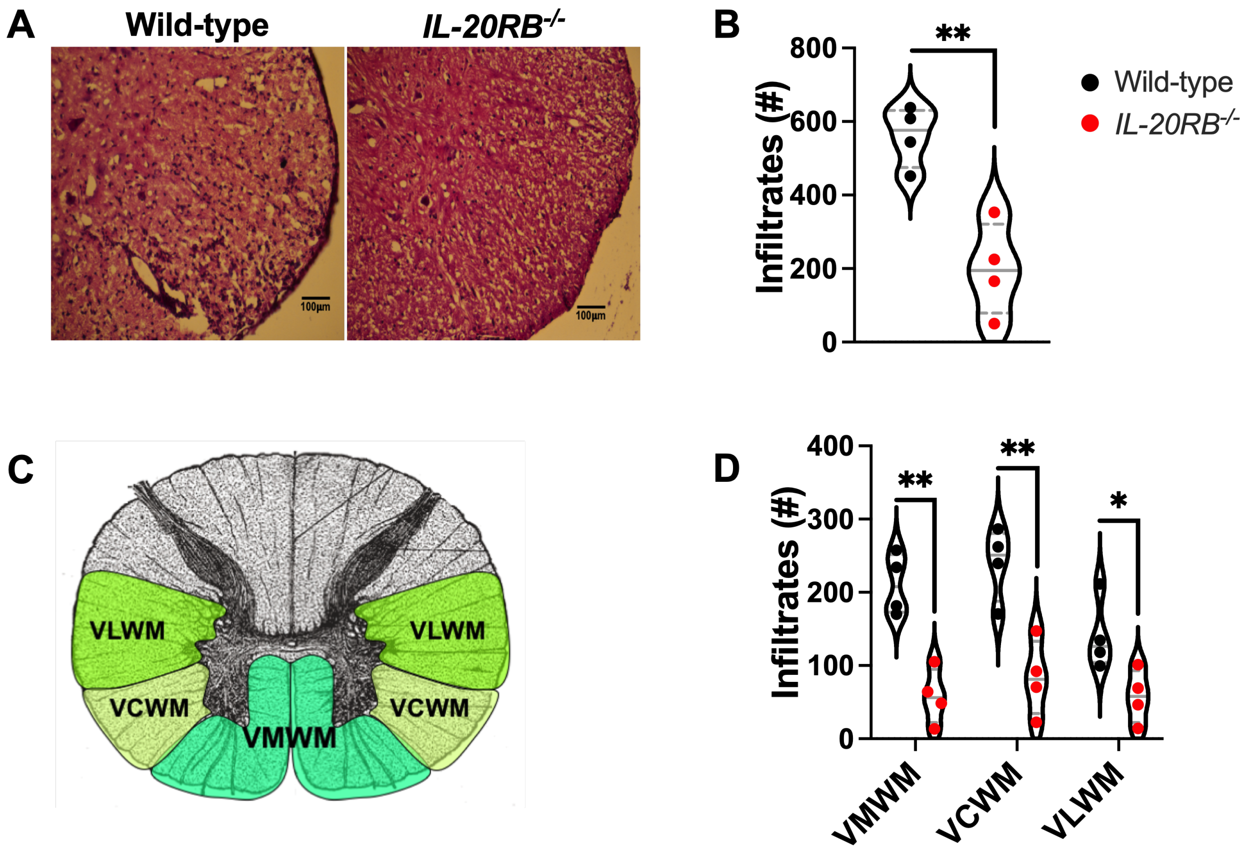

Supplemental Figure 1. *IL-20RB$^{-/-}$* mice exhibits reduction of perivascular infiltrates. *IL-20RB$^{-/-}$* mice and wild-type mice were actively immunized for EAE and euthanized at peak (~14 dpi) for histological analysis, n = 4. H&E staining was performed on spinal cords, following standardized protocols (representative image, scale bar = 100 µm, **A**). Means of total number of perivascular infiltrates throughout ventral white matter were analyzed by unpaired t-test showed statistical significance between wild-type and *IL-20RB$^{-/-}$* mice (mean ± SEM: 560.4 ± 41.15 vs 198.4 ± 63.06) p <0.01 **(B)**. Perivascular infiltrates were also analyzed based on location: ventromedial white matter, VMWM; ventral column white matter, VCWM and ventrolateral white matter, VLWM **(C).** Comparison of perivascular infiltrates between wild-type and *IL-20RB$^{-/-}$* mice and analysis by Two-Way ANOVA analysis followed by Sidak's post hoc test showed statistical significance for VMWM (mean ± SEM: 210.75 ± 20.90.15 vs 57.75 ± 18.96), p <0.01, VCWM (mean ± SEM: 239.63 ± 24.96.15 vs 83.00 ± 25.81), and VLWM (mean ± SEM: 140.88 ± 24.60 vs 57.63 ± 18.34), p <0.05 **(D)**. Results are shown as Mean ± SEM, * = p <0.05 and ** = p <0.01.
