# Supplementary material for: Expression of IL-20 Receptor Subunit β Is Linked to EAE Neuropathology and CNS Neuroinflammation
Source: Front Cell Neurosci. 2021 Sep 7;15:683687. doi: 10.3389/fncel.2021.683687 (PMC8452993; doi:10.3389/fncel.2021.683687)
Supplement: Supplementary Figure 2 — Heterozygosity of IL-20 does not alter EAE disease progression. Ten week-old IL-20–/– mice, male and female, were actively immunized for EAE (red circle) or sham-immunized (black circle) as control (n = 10). Clinical score, body weight, highest and cumulative scores, from 7 to 28 dpi were analyzed by unpaired t-test (A–C). Difference in clinical score and body weight, highest and cumulative scores, between actively immunized IL-20+/– mice and control showed extreme statistical significance, p < 0.0001 (mean ± SEM clinical score: 1.14 ± 0.18 vs. 0.00 ± 0.00, body weight: 20.59 ± 0.14 vs. 22.28 ± 0.15, A) (mean ± SEM highest score: 2.95 ± 0.38 vs. 0.00 ± 0.00, B) (mean ± SEM cumulative score: 22.50 ± 3.41 vs. 0.00 ± 0.00, C). Disease onset of actively immunized IL-20+/– mice with neurologic symptoms (10/10, mean ± SEM: 14.20 ± 0.42) (D). Analysis of percentage of survival was not deemed significant (E). Results are shown as mean ± SEM, ****p < 0.0001. [file Image_2.pdf]

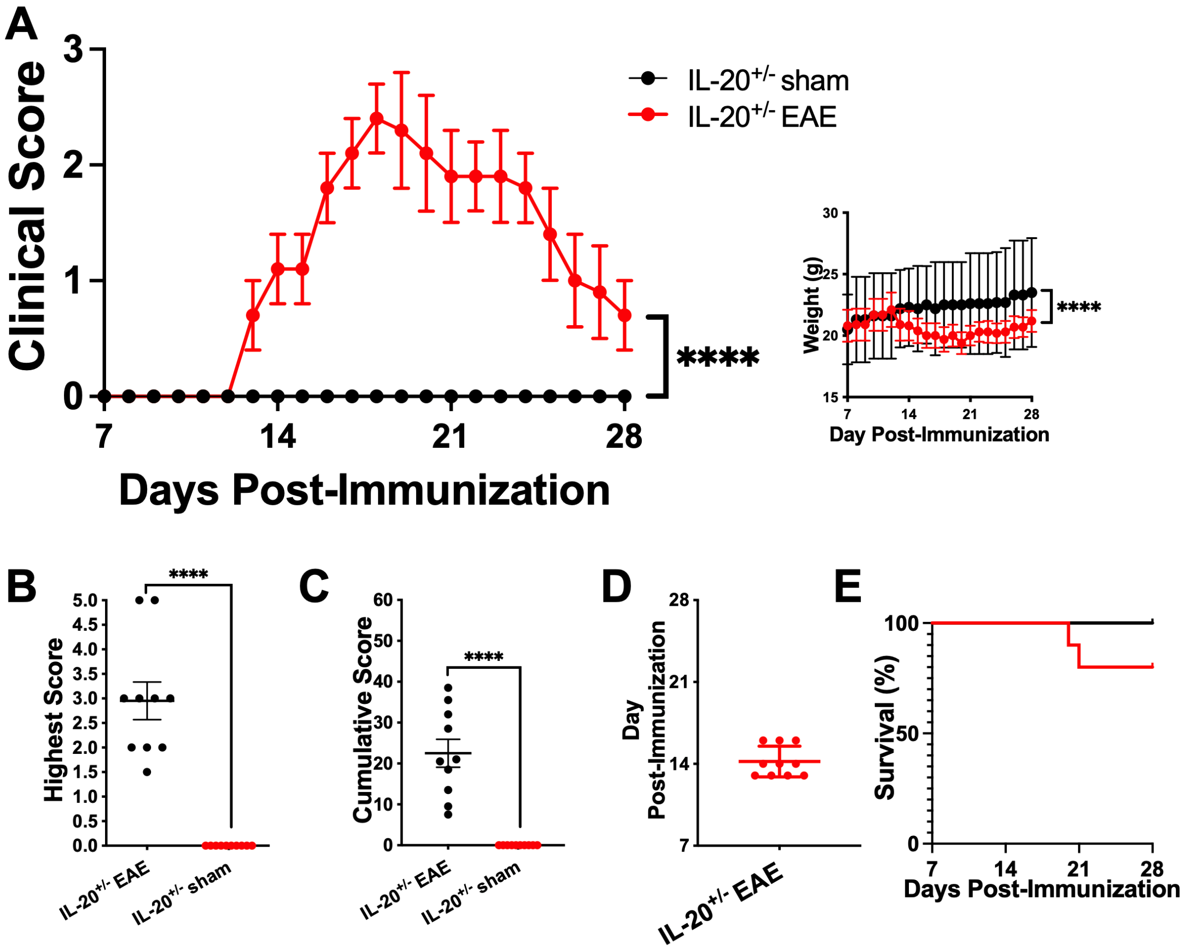

Supplemental Figure 2. Heterozygosity of IL-20 does not alter EAE disease progression. Ten week-old *IL-20*$^{+/-}$ mice, male and female, were actively immunized for EAE (red circle) or sham-immunized (black circle) as control (n = 10). Clinical score, body weight, highest and cumulative scores, from 7 dpi to 28 dpi were analyzed by unpaired t-test **(A-C).** Difference in clinical score and body weight, highest and cumulative scores, between actively immunized *IL-20*$^{+/-}$ mice and control showed extreme statistical significance, $p<0.0001$ (mean ± SEM clinical score: 1.14 ± 0.18 vs 0.00 ± 0.00, body weight: 20.59 ± 0.14 vs 22.28 ± 0.15, **A**) (mean ± SEM highest score: 2.95 ± 0.38 vs 0.00 ± 0.00, **B**) (mean ± SEM cumulative score: 22.50 ± 3.41 vs 0.00 ± 0.00, **C**). Disease onset of actively immunized *IL-20*$^{+/-}$ mice with neurologic symptoms (10/10, mean ± SEM: 14.20 ± 0.42) **(D)**. Analysis of percentage of survival was not deemed significant **(E)**. Results are shown as Mean ± SEM, **** = $p <0.0001$.
