# Supplementary material for: Expression of IL-20 Receptor Subunit β Is Linked to EAE Neuropathology and CNS Neuroinflammation
Source: Front Cell Neurosci. 2021 Sep 7;15:683687. doi: 10.3389/fncel.2021.683687 (PMC8452993; doi:10.3389/fncel.2021.683687)
Supplement: Supplementary Figure 3 — Necropsy of sham-immunized mice treated with MABIL-20 and IgG2B. C57BL6/J mice, male and female, n = 4, were treated with 10 mg/kg BW of rat monoclonal anti-IL-20 (MABIL-20) and IgG2B isotype control, 10 days after sham immunization to assess safe use of monoclonal antibody. Treatment continued for 10 days, then mice were euthanized for histological analysis. Comparison between MABIL-20 and IgG2B tissues were unremarkable. Representative images for brain (A), skin (B), lung (C), knee (D), spleen (E), cervical, axillary, inguinal lymph nodes (F–H, respectively), kidney (I), liver (J), stomach (K), small and large intestine (L,M, respectively). [file Image_3.pdf]

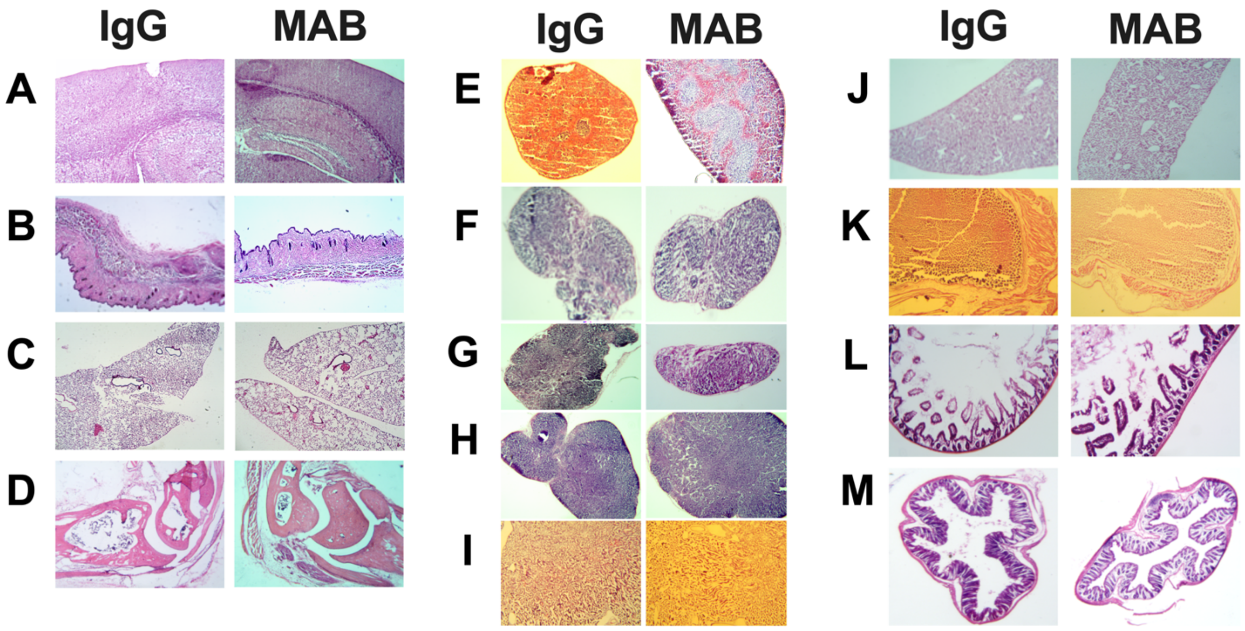

Supplemental Figure 3. Necropsy of sham-immunized mice treated with MABIL-20 and IgG_{2b}. C57BL6/J mice, male and female, n = 4, were treated with 10 mg/kg BW of rat monoclonal anti-IL-20 (MABIL-20) and IgG_{2B} isotype control, 10 days after sham immunization to assess safe use of monoclonal antibody. Treatment continued for 10 days, then mice were euthanized for histological analysis. Comparison between MABIL-20 and IgG_{2B} tissues were unremarkable. Representative images for brain **(A),** skin **(B)**, lung **(C)**, knee **(D)**, spleen **(E)**, cervical, axillary, inguinal lymph nodes (**F-H**, respectively), kidney **(I)**, liver **(J)**, stomach **(K)**, small and large intestine (**L, M**, respectively).
