# Supplementary material for: Expression of IL-20 Receptor Subunit β Is Linked to EAE Neuropathology and CNS Neuroinflammation
Source: Front Cell Neurosci. 2021 Sep 7;15:683687. doi: 10.3389/fncel.2021.683687 (PMC8452993; doi:10.3389/fncel.2021.683687)
Supplement: Supplementary Figure 4 — Neutralization of IL-20 cytokine has no impact on clinical progression of EAE. Ten week-old C57BL6/J mice, male and female, were actively immunized for EAE, sham-immunized (not show) as control (EAE, n = 12; sham, n = 4). Treatment with 10 mg/kg BW of rat monoclonal anti-IL-20 (MABIL-20) and IgG2B isotype control started when mice reached a score or 2 (hindlimb paresis). Clinical score, body weight, highest and cumulative scores from 7 to 35 dpi were analyzed by unpaired t-test and changes were not deemed significant (A–C). Analysis of survival curve was also not significant (D). Results are shown as mean ± SEM. [file Image_4.pdf]

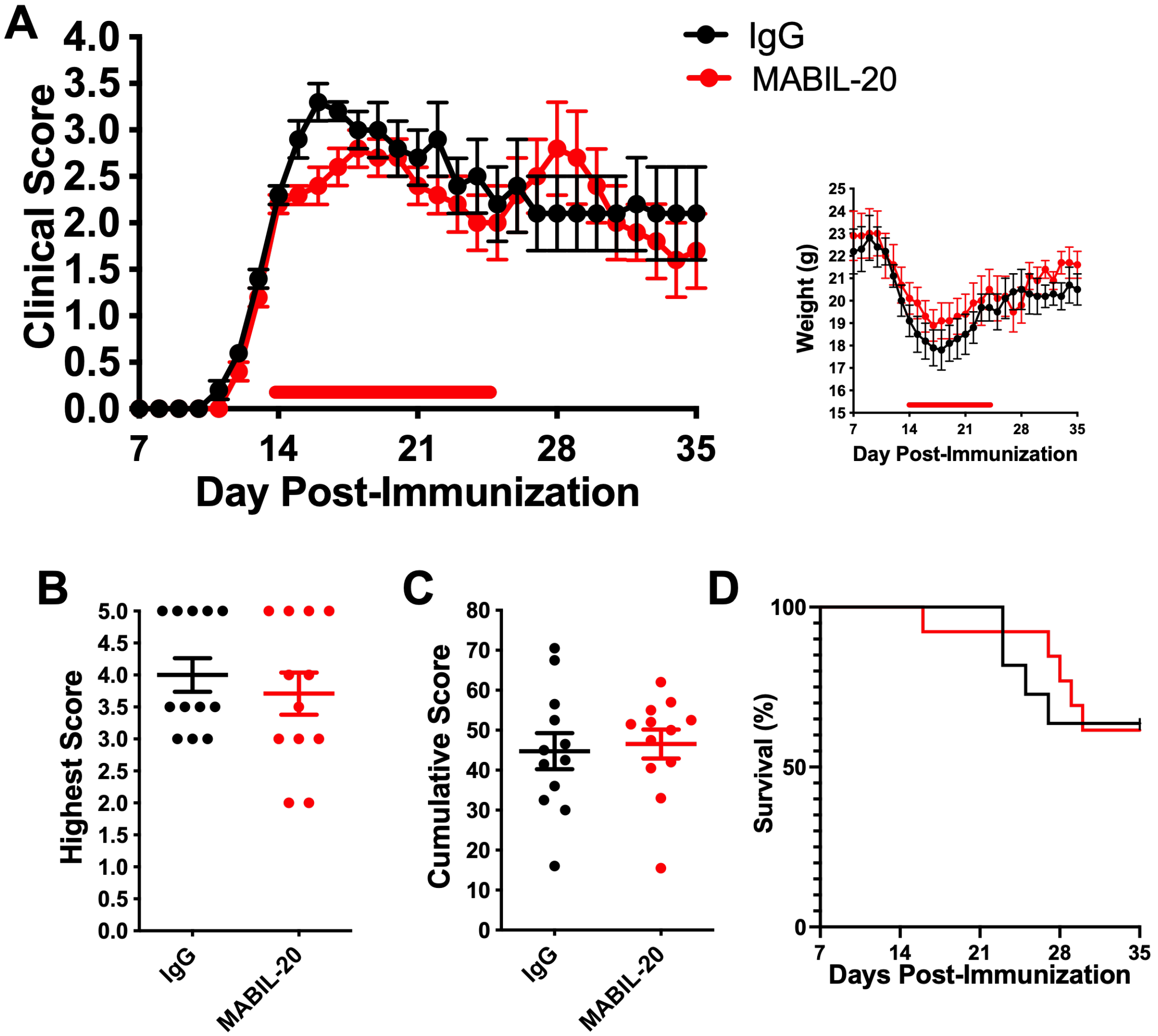

Supplemental Figure 4. Neutralization of IL-20 cytokine has no impact on clinical progression of EAE. Ten week-old C57BL6/J mice, male and female, were actively immunized for EAE, sham-immunized (not show) as control (EAE, n = 12; sham, n = 4). Treatment with 10 mg/kg BW of rat monoclonal anti-IL-20 (MABIL-20) and IgG_{2B} isotype control started when mice reached a score or 2 (hindlimb paresis). Clinical score, body weight, highest and cumulative scores from 7 dpi to 35 dpi were analyzed by unpaired t-test and changes were not deemed significant **(A-C)**. Analysis of survival curve was also not significant **(D)**. Results are shown as Mean ± SEM.
