# Supplementary material for: Expression of IL-20 Receptor Subunit β Is Linked to EAE Neuropathology and CNS Neuroinflammation
Source: Front Cell Neurosci. 2021 Sep 7;15:683687. doi: 10.3389/fncel.2021.683687 (PMC8452993; doi:10.3389/fncel.2021.683687)
Supplement: Supplementary Figure 7 — Cytokine expression at the brainstem of wild-type mice and IL-20RB–/– mice at peak of EAE. Cytokines and VEGF expression within brainstem tissues were detected by multianalyte bead-based immunoassay (A–M) and ELISA (N–P), n = 4 (same cohort of mice shown on Figure 3 and Supplementary Figure 5), for wild-type mice at peak of EAE (peak, ∼14 dpi) (black circle) and sham-immunized, IL-20RB–/– mice (red circle), and sham-immunized (sham-immunized not shown). One-way ANOVA analysis followed by Sidak’s post hoc comparing wild-type mice and IL-20RB–/– mice at peak of EAE showed statistical significance for GM-CSF, p < 0.01 (J) and TNF-α, p < 0.001 (L). Results are shown as mean ± SEM, ∗p < 0.05 and ∗∗∗p < 0.001. [file Image_7.pdf]

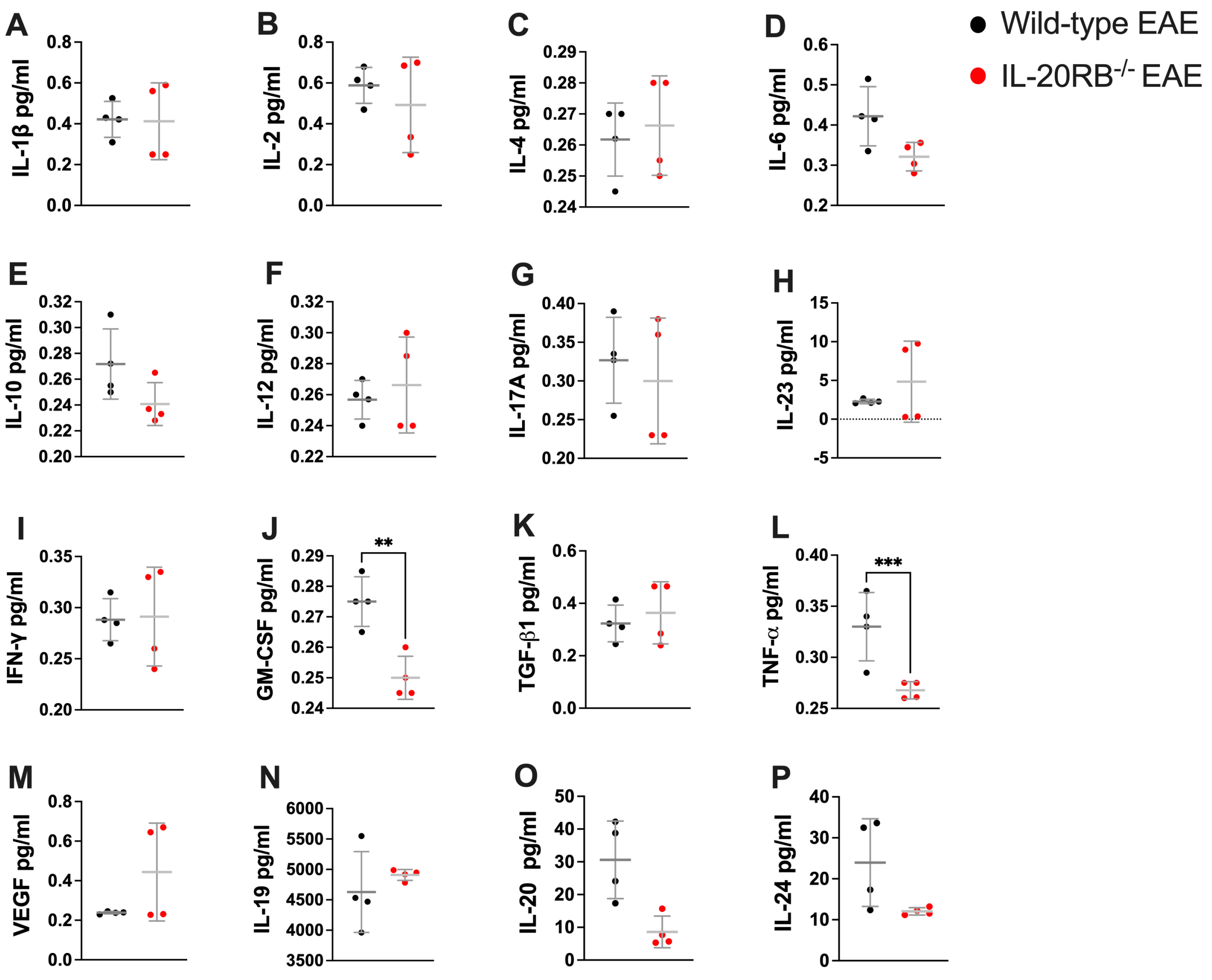

Supplemental Figure 7. Cytokine expression at the brainstem of wild-type mice and *IL-20RB$^{-/-}$* mice at peak of EAE. Cytokines and VEGF expression within brainstem tissues were detected by multianalyte bead-based immunoassay **(A-M)** and ELISA **(N-P)**, n = 4 (same cohort of mice shown on Figure 3 and Supplemental Figure 5), for wild-type mice at peak of EAE (peak, ~14 dpi) (black circle) and sham-immunized, *IL-20RB$^{-/-}$* mice (red circle) and sham-immunized (sham-immunized not shown). One-Way ANOVA analysis followed by Sidak's post hoc comparing wild-type mice and *IL-20RB$^{-/-}$* mice at peak of EAE showed statistical significance for GM-CSF, $p < 0.01$ **(J)** and TNF-α, $p < 0.001$ **(L)**. Results are shown as Mean ± SEM, * = $p < 0.05$ and *** = $p < 0.001$.
