# Supplementary material for: Expression of IL-20 Receptor Subunit β Is Linked to EAE Neuropathology and CNS Neuroinflammation
Source: Front Cell Neurosci. 2021 Sep 7;15:683687. doi: 10.3389/fncel.2021.683687 (PMC8452993; doi:10.3389/fncel.2021.683687)
Supplement: Supplementary Figure 9 — Murine expression of IL-20 receptor subunits. Murine microvessels were isolated from naïve C57BL/6J mice, n = 3, to elucidate IL-20 receptor subunits protein expression by immunolabeling for IL-22RA1 (white), IL-20RB (red), IL-20RA (green), and DAPI (blue), scale bar = 10 μm (A). Histogram analysis was performed to 25 microvessels per specimen to determine AUI and AUC of each receptor subunit (representative AUI vs. axis plot histogram, B). Although AUC means exhibit a trend IL-22RA1 > IL-20RB > IL-20RA, one-way ANOVA analysis followed by Sidak’s post hoc test showed that mean AUC differences between subunits are not statistically significant (mean ± SD IL-22RA1: 6.2 × 10^–4^ ± 2.2 × 10^–4^, IL-20RB: 4.2 × 10^–4^ ± 1.7 × 10^–4^, and IL-20RA: 5.4 × 10^–4^ ± 2.1 × 10^–4^, C). Results are shown as mean ± SD. [file Image_9.pdf]

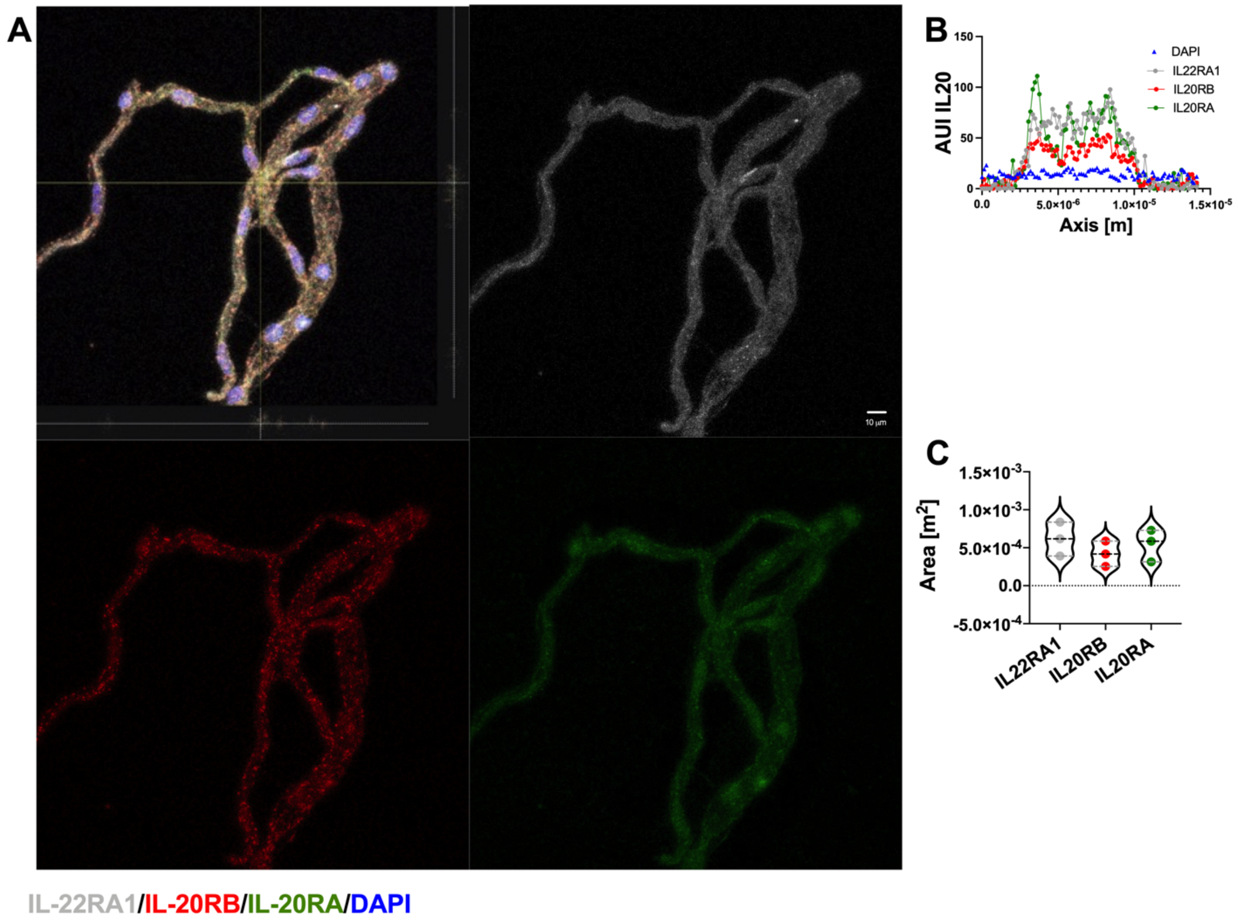

Supplemental Figure 9. Murine expression of IL-20 receptor subunits. Murine microvessels were isolated from naïve C57BL/6J mice, n = 3, to elucidate IL-20 receptor subunits protein expression by immunolabeling for IL-22RA1 (white), IL-20RB (red), IL-20RA (green), and DAPI (blue), scale bar = 10 µm **(A)**. Histogram analysis was performed to 25 microvessels per specimen to determine AUI and AUC of each receptor subunit (representative AUI vs axis plot histogram, **B**). Although AUC means exhibit a trend IL-22RA1 > IL-20RB > IL-20RA, One-Way ANOVA analysis followed by Sidak's post hoc test showed that mean AUC differences between subunits are not statistically significant (mean ± SD IL-22RA1: $6.2 \times 10^{-4} \pm 2.2 \times 10^{-4}$, IL-20RB: $4.2 \times 10^{-4} \pm 1.7 \times 10^{-4}$, and IL-20RA: $5.4 \times 10^{-4} \pm 2.1 \times 10^{-4}$, **C**). Results are shown as Mean ± SD.
